# Iron oxides nanoparticles (IOs) exposed to magnetic field promote expression of osteogenic markers in osteoblasts through integrin alpha-3 (INTa-3) activation, inhibits osteoclasts activity and exerts anti-inflammatory action

**DOI:** 10.1186/s12951-020-00590-w

**Published:** 2020-02-18

**Authors:** K. Marycz, P. Sobierajska, M. Roecken, K. Kornicka-Garbowska, M. Kępska, R. Idczak, J.-M. Nedelec, R. J. Wiglusz

**Affiliations:** 1 The Department of Experimental Biology, University of Environmental and Life Sciences Wroclaw, Norwida 27B, 50-375 Wrocław, Poland; 2 Faculty of Veterinary Medicine, Equine Clinic-Equine Surgery, Justus-Liebig-University, Frankfurter 108, 35392 Giessen, Lahn, Germany; 3 Institute of Low Temperature and Structure Research, Polish Academy of Sciences, Okolna 2, 50-422 Wrocław, Poland; 4 International Institute of Translational Medicine, Jesionowa 11, Malin, 55-114 Wisznia Mała, Poland; 5 Université Clermont Auvergne, CNRS, SIGMA Clermont, ICCF, Clermont-Ferrand, France; 6 Centre for Advanced Materials and Smart Structures, Polish Academy of Sciences, Okolna 2, 50-950 Wrocław, Poland

**Keywords:** Iron oxides, Osteoblasts, Osteoclasts, Osteoporosis

## Abstract

**Background:**

Prevalence of osteoporosis is rapidly growing and so searching for novel therapeutics. Yet, there is no drug on the market available to modulate osteoclasts and osteoblasts activity simultaneously. Thus in presented research we decided to fabricate nanocomposite able to: (i) enhance osteogenic differentiation of osteoblast, (i) reduce osteoclasts activity and (iii) reduce pro-inflammatory microenvironment. As a consequence we expect that fabricated material will be able to inhibit bone loss during osteoporosis.

**Results:**

The α-Fe_2_O_3_/γ-Fe_2_O_3_ nanocomposite (IOs) was prepared using the modified sol–gel method. The structural properties, size, morphology and Zeta-potential of the particles were studied by means of XRPD (X-ray powder diffraction), SEM (Scanning Electron Microscopy), PALS and DLS techniques. The identification of both phases was checked by the use of Raman spectroscopy and Mössbauer measurement. Moreover, the magnetic properties of the obtained IOs nanoparticles were determined. Then biological properties of material were investigated with osteoblast (MC3T3), osteoclasts (4B12) and macrophages (RAW 264.7) in the presence or absence of magnetic field, using confocal microscope, RT-qPCR, western blot and cell analyser. Here we have found that fabricated IOs: (i) do not elicit immune response; (ii) reduce inflammation; (iii) enhance osteogenic differentiation of osteoblasts; (iv) modulates integrin expression and (v) triggers apoptosis of osteoclasts.

**Conclusion:**

Fabricated by our group α-Fe_2_O_3_/γ-Fe_2_O_3_ nanocomposite may become an justified and effective therapeutic intervention during osteoporosis treatment.

## Background

In recent years, regenerative medicine as well as theranostics are rapidly developing fields of medicine. This new branch of medical sciences targets not only regeneration of damage tissues, but also attempts to visualize regeneration process in vivo. The role of these research areas is especially profound in studying of bone diseases which nowadays requires interdisciplinary approach combining basic, clinical and translational studies. Due to progressive aging of population, the bone tissue is affected by deterioration of its composition, structure and function [1]. All of that predispose to the development of osteoporosis, disease defined by massive bone loss and significant changes in bone microarchitecture. Decreased biomechanical properties and impaired young modulus of osteoporotic bones contribute to great risk of fractures. According to the definition of osteoporosis introduced by World Health Organization (WHO), osteoporotic patient exhibits reduction in bone mineral density (BMD) of 2.5 standard deviations or more, below that of the mean peak BMD of young adults when measured by dual-energy X-ray absorptiometry (DEXA) [2]. As reported by Food and Drug Administration (FDA) only in United States of America currently near 10 million of patients suffer from osteoporosis, contributing to economic burden on the health care system [3]. The estimated cost of osteoporosis management is estimated an average of $17–$20 billion per year [4]. That enormous numbers highlight the need to develop a novel, effective therapeutic intervention and fully unravel the molecular mechanisms leading to disease development.

The imbalance between the bone formation, orchestrated by bone-forming cells (osteoblasts and osteocytes) and bone resorption are recognized as a main reason of osteoporosis development [5]. Thus restoring the proper interaction between osteoblasts and osteoclasts may become a target for a new drug generation. Modulation of osteoblast viability and enhancement of their differentiation potential become one of the fundamental factors in the course of bone regeneration. During recent years, multiple strategies to counter bone loss have been developed and tested. Pharmacological treatment including application of drugs aims to inhibit bone resorption and promote bone formation (i.e. bisphosphonates and teriparatide respectively). There has been also interest in looking at combination therapies to simultaneously modulate two processes [6]. On the other hand, there are also attempts to increase fracture healing in osteoporosis patients which include development of biomaterial scaffolds [7] and modification of existing bone surfaces to promote native bone growth [8]. Although substantial improvements in the treatment of osteoporosis during recent years, osteoporotic fractures are still a major clinical challenge in especially in the elderly due to impaired healing. Thus fabrication of smart biomaterials able to modulate bone cells fate and re-establish normal bone repair is absolutely necessary to diminish economic burden of the disease.

Recent data have indicated that not only osteoclasts and osteoblasts participate in the osteoporosis pathogenesis but also certain types of immune cells including T cells, B cells and macrophages [9]. Activated macrophages secrete wide range of proinflammatory cytokines which induce osteoclastogenesis and bone loss [9]. Thus, modulation of macrophages activity has become interesting alternative for the inhibition of bone resorption in osteoporotic patients. That thesis is supported by the recent research performed by Rao et al. [10] who discovered that downregulation of pro-inflammatory cytokines prevents inflammation induced osteoporosis. Also, macrophages directly participate in the formation and activation of osteoclasts [11]. On the other hand, maintaining physiological activity of macrophages is necessary to bone formation, since it was shown, that macrophages depletion in mice resulted in 25% reduction of bone mineral density and a 70% reduction in the number of trabecular bone compared to control littermates [12].

The bone formation and bone resorption processes might be mediated by external factors including magnetic iron oxide nanoparticles (IOs) [13, 14]. Ferrite nanoparticles belong to the most common applied magnetics nanoparticles for biomedical applications [15]. Magnetic properties of IOs make them a promising factor in the development of new drugs or gene delivery systems. The possibility to track IOs using magnetic resonance imaging (MRI) opens potential application of IOs based biomaterials in clinics, especially as they were proved to be chemically stable and nontoxic in vivo [16]. Recently, it has been shown that hydroxyapatite and IOs in the chitosan-based composites promote new bone formation [17] which supports their application in osteoporosis. Furthermore, their unique properties find wide applications, since coated IOs under magnetic field gradient are successfully carried to the desired site with relatively high accuracy [18]. This results in significant reduction of necessary surgical intervention, application of maximum dose of drugs, and avoidance of toxic side effects on other organs. Our own previous studies revealed that magnetic promote osteogenic differentiation potential of stem progenitor cells under magnetic field condition through activation of particular integrin’s [19].

In this study, for the first time we investigated the effects of IOs on osteoblasts and osteoclasts activity. Moreover, we performed the co-culture tests using macrophages to verify if IOs in magnetic field promotes M1 or either M2 macrophages polarization. As IOs can be easily synthesizable and incorporate into selected scaffolds, they can be applied in multiple biomedical applications including the treatment of osteoporosis. On this basis, we have investigated the ability of IOs to induce osteoinduction and inhibit osteoclasts activity. Remarkably, obtained particles enhanced expression of pro-osteogenic genes in osteoblasts while at the same time triggered osteoclast apoptosis. Our findings show for the first time that fabricated by our group IOs can be applied in the fabrication of bone regeneration scaffolds for osteoporotic patients due to their in vitro cytocompatibility and ability to modulate osteoblasts, osteoclasts and macrophages properties.

## Results

### Physicochemical characterisation of IOs nanoparticles

**Fig. 1:**
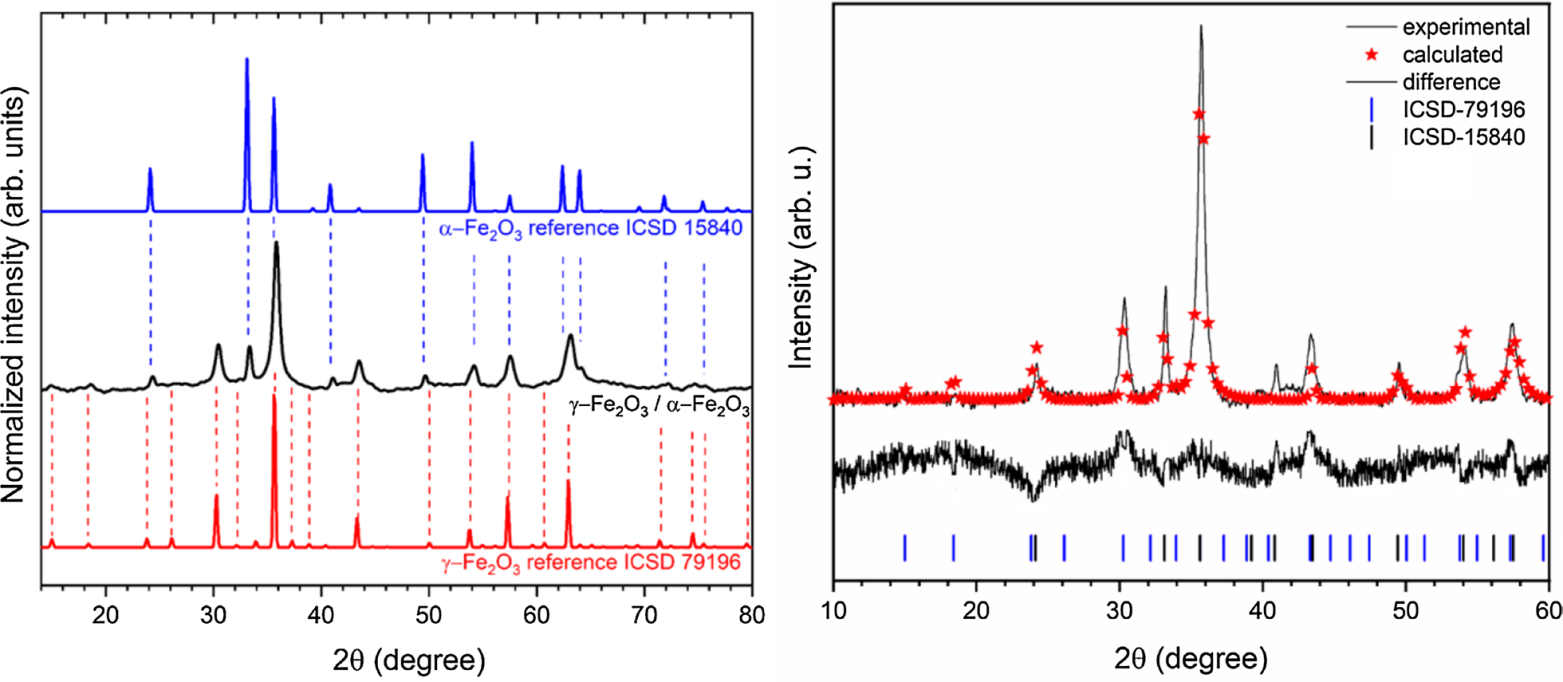
X-ray powder diffraction pattern of the iron oxide powder (left), prepared by modified sol–gel method and annealed at 300 °C, in relation to the position of reference peaks originated from alpha and gamma phases. Result of the Rietveld analysis (right) of the nanocomposite α-Fe_2_O_3_/γ-Fe_2_O_3_ heated at 300 °C (black line– XRD pattern; red—fitted diffraction; black—differential pattern; blue and black—positions of reference phase peaks)

Crystal structure of the obtained IOs was measured using XRPD technique. The presented XRPD patterns in Fig. 1 (left) show that all diffraction peaks overlap with two iron oxide phases—cubic crystal structure of γ-Fe_2_O_3_ (maghemite) and trigonal crystal structure of α-Fe_2_O_3_ (hematite). No additional phases, impurities or amorphous forms were detected. It should be mentioned that the XRPD patterns of γ-Fe_2_O_3_ and Fe_3_O_4_ (magnetite) are very similar. However, the synthesis route of material under air condition as well as excluding the iron ion source on the second oxidation state (Fe^2+^) leads to the conclusion that one of the product obtained is γ-Fe_2_O_3_. Hematite crystallizes in the R-3c lattice system with two-thirds of the octahedral sites occupied by Fe^3+^. While, maghemite is a structure based on 32 O^2−^, 21-1/3 Fe^3+^, and 21/3 vacancies. The characteristic peak from the hematite is clearly visible at 33.2°, while peak at 35.7° overlaps with those from the maghemite. The contribution of these two phases in the final product and their cell parameters were calculated using Rietveld analysis in isotropic approach applying Maud 2.91 software. The quality of structural refinement for α-Fe_2_O_3_ and γ-Fe_2_O_3_ was checked by *R* values. The parameters with additional functions were applied to obtain a structural refinement with better quality and reliability. The confirmation of the trigonal (α-Fe_2_O_3_—ICSD 15840) [20] and cubic (γ-Fe_2_O_3_—ICSD 79196) [21] phase formations was affirmed by the results. Figure 1 (right) shows a good relationship between the observed XRPD pattern and the theoretical fit which indicates the validity of the Rietveld refinement as illustrated by the close to zero differences in the intensity scale in the line (Y_Obs_ − Y_Calc_). More details regarding Rietveld refinement have been shown in Table 1. However, the crystallite size was estimated to be 37 nm  for α-Fe_2_O_3_ and 88 nm for γ-Fe_2_O_3_. It is worth noting that the phase content was calculated to be 15.34% α-Fe_2_O_3_ and 84.66% for γ-Fe_2_O_3_.

**Table 1:** Unit cell parameters (a, c), crystal cell volume (V), as well as refined factor (R_w_) for the nanocomposite α-Fe_2_O_3_/γ-Fe_2_O_3_

Cell parameters						Phase		
Sample	Hematite α-Fe_2_O_3_			Maghemite γ-Fe_2_O_3_		α-Fe_2_O_3_ (%)	γ-Fe_2_O_3_ (%)	R_w_
	a (Å)	c (Å)	V (Å^3^)	a (Å)	V (Å^3^)			
s.c.	5.038 (2)	13.772 (1)	302.72 (3)	8.3474 (2)	581.64 (2)	**–**	**–**	–
Composite	5.037 (1)	13.760(0)	302.35 (6)	8.3520 (8)	582.61 (8)	15.34	84.66	0.86

*s. c.* single crystal reference data, α-Fe_2_O_3_—ICSD 15840, γ-Fe_2_O_3_—ICSD 79196

**Fig. 2:**
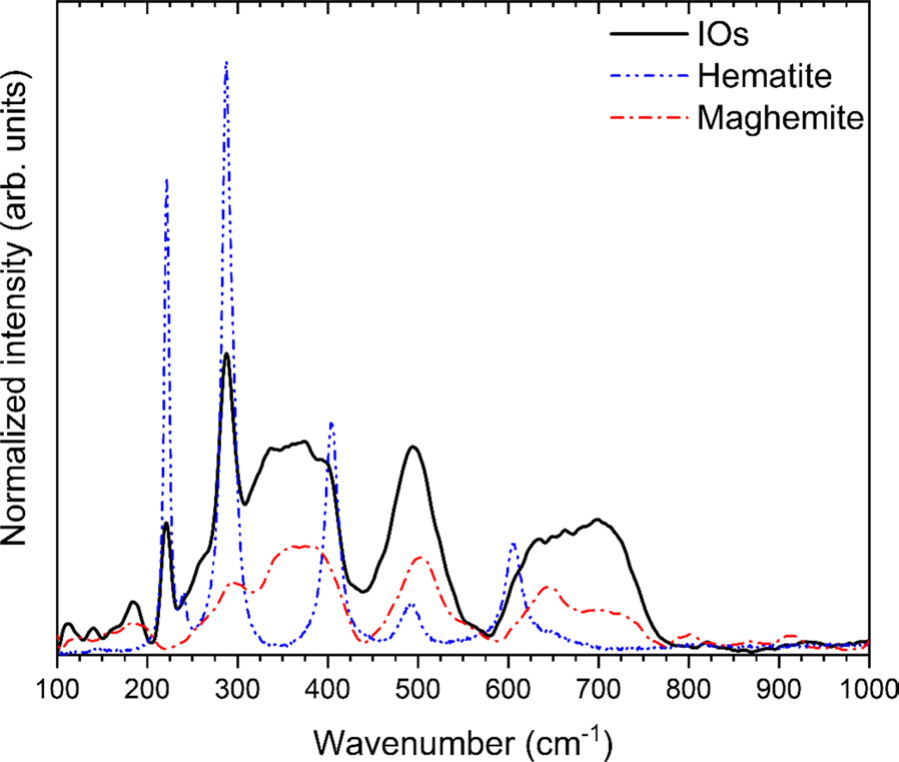
Raman spectrum of IOs in comparison with characteristic spectrum of hematite (blue line) and maghemite (red line)

In this paper, Raman microscopy was also employed to identified the studied iron oxide products. The spectrum of IOs was obtained directly from powdered samples with very low laser power (0.9 mW) in order to minimize the problems caused by phase transitions under higher laser power irradiation. The obtained spectrum of IOs was compared with characteristic spectrum of hematite (blue line) and maghemite (red line) (see Fig. 2). Some of the peaks belonged to the observed spectrum are similar to that of well-known hematite [22]. The most characteristic peaks of α-Fe_2_O_3_ are located at 221, 288 cm^−1^. Additional peaks at 240, 405, 493, 605 cm^−1^ are overlapped with those originated from other phase of iron oxide. Three broad bands located around 350, 500 and 700 cm^−1^ have been identified for maghemite structure, not observed in any other spectrum of iron oxide [22].

**Fig. 3:**
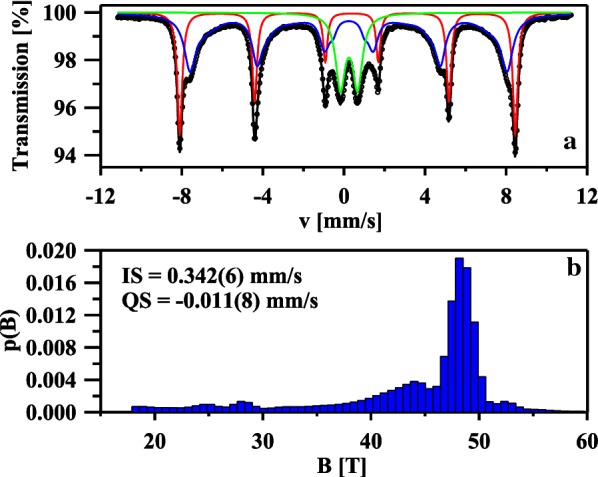
The room-temperature ^57^Fe Mössbauer spectrum of the nanocomposite α-Fe_2_O_3_/γ-Fe_2_O_3_ fitted using one sextet (red component), one distribution of hyperfine fields (blue component) and one doublet (green component) (**a**). The hyperfine magnetic field distribution of the blue component (**b**)

The identification of both phases and their relative proportions were further checked by the use of Mössbauer measurements. Figure 3a shows the room-temperature ^57^Fe Mössbauer spectrum of the nanocomposite α-Fe_2_O_3_/γ-Fe_2_O_3_. The fitting was made using one magnetic six-line pattern (sextet), one quadrupole split doublet and one distribution of hyperfine fields. The sextet with B = 51.3(1) T, IS = 0.390(2) mm/s, QS = − 0.211(3) mm/s and Γ = 0.26(1) mm/s corresponds to well-crystallized α-Fe_2_O_3_ nanoparticles with the average grain size greater than 12 nm [23, 24]. The second magnetic component could be described by IS = 0.342(6) mm/s, QS = − 0.011(8) mm/s and the hyperfine magnetic field distribution which is presented in Fig. 3b. According to the previous Mössbauer studies of maghemite nanopowders [25–27], that component is connected with γ-Fe_2_O_3_ nanoparticles with various grain sizes. As one can notice in Fig. 3b, the p(B) distribution has a pronounced maximum around 48.5 T. This maximum could be attributed to the Fe atoms located in the bulk of larger γ-Fe_2_O_3_ nanoparticles while the smaller maxima at B < 48.5 T correspond to iron atoms in the surface layer of these nanoparticles [25, 27] At the same time, the quadrupole split doublet with IS = 0.356(5) mm/s, QS = 0.84(1) mm/s and Γ = 0.55(1) mm/s is connected with Fe atoms in ultrafine superparamagnetic γ-Fe_2_O_3_ nanoparticles [25–27]. The values of the Mössbauer hyperfine parameters, in particular the IS values determined for all three components are typical of ferric Fe^3+^ ions and no ferrous Fe^2+^ ions were found in the sample. This indicates that the magnetite Fe_3_O_4_ phase is not present in prepared nanoparticles and that is in agreement with XRPD and Raman measurements. Finally, the relative proportions of both Fe_2_O_3_ phases are estimated. The spectral area fractions for the α-Fe_2_O_3_, magnetically ordered γ-Fe_2_O_3_ and superparamagnetic γ-Fe_2_O_3_ phases are 29.6(1)%, 54.1(2)% and 18.5(1)%, respectively.

**Fig. 4:**
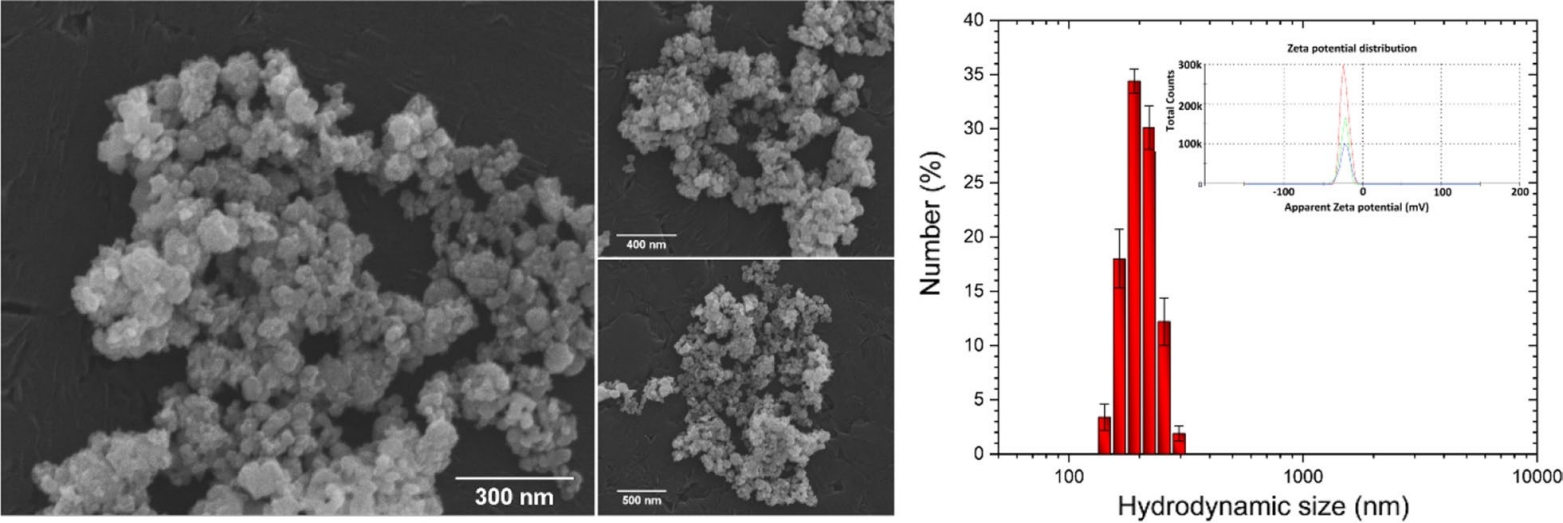
SEM images of the IOs with the indication of nano-sized grains (left). Main hydrodynamic size and Zeta potential (inset) of IOs suspension (right)

In order to characterize the morphological properties of the obtained IOs nanoparticles SEM–EDS analysis was performed. As can be seen in the SEM images (Fig. 4, left), the particles possess the nanocrystalline form (their size is much less than 100 nm) with relatively homogeneous distribution and show tendency to aggregate into spherical-like objects. In biological point of view, the most valuable results of the grain size can be given by DLS technique. The particle dispersed in a liquid media are characterized by hydrodynamic size which is often larger than the primary particle size (see SEM images). The hydrodynamic radius (*r*_*h*_) of the studied IOs has been determined using the Stokes–Einstein equation:

$$r_{h} = \frac{{K_{B} T}}{{6 \pi \eta D_{t} }}$$

where *K*_*B*_ is Boltzmann’s constant, *T* is temperature, *D*_*t*_ is particle diffusion coefficient and *η* is solvent viscosity (H_2_O). The results were gathered in the diagram (Fig. 4, right)) and as it was shown, typical hydrodynamic size of the studied IOs was in the range 190–220 nm. Moreover, the IOs nanoparticles in water suspension were negatively charged with Zeta potential equal – 21.50 mV.

**Fig. 5:**
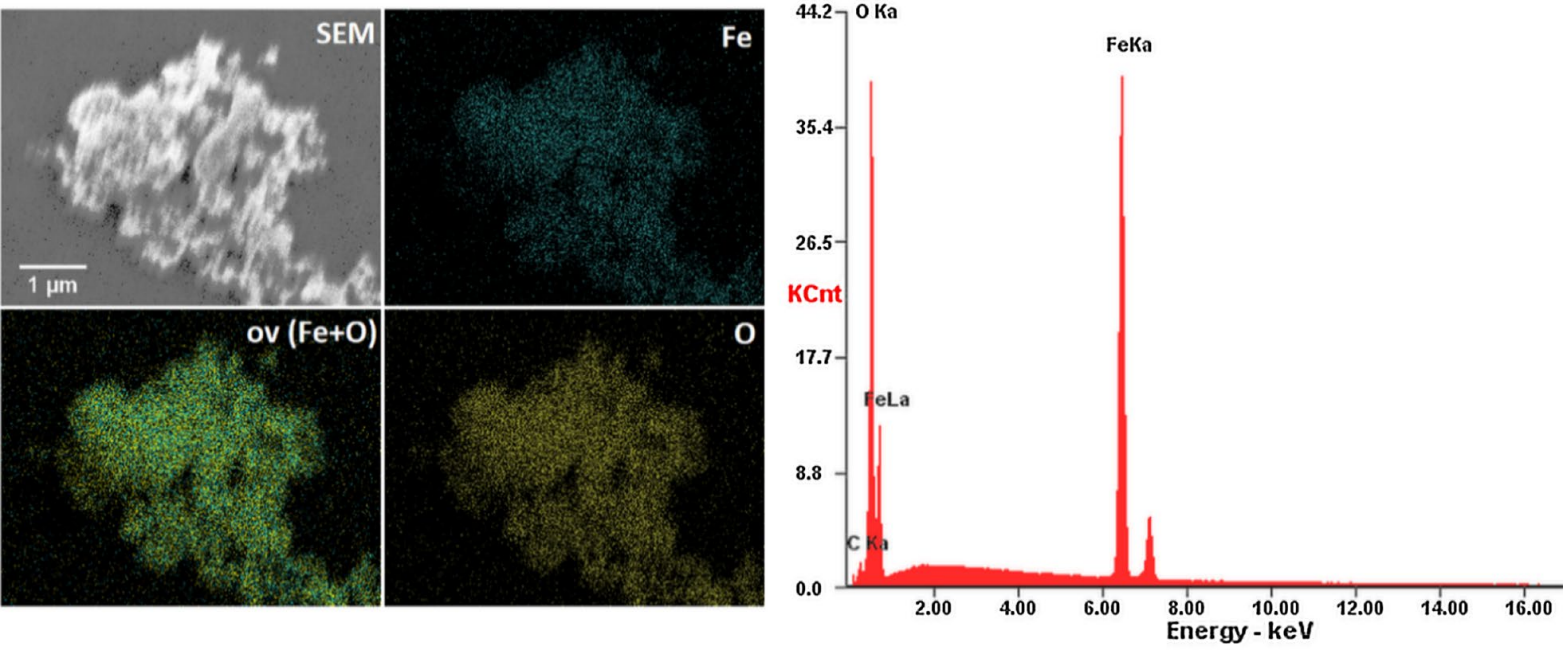
SEM-EDS elemental maps (left) and EDS spectrum (right) of the IOs nanopowder

Moreover, the SEM–EDS elemental maps of Fe and O (including overlapped maps) have been measured. It is clearly visible that both iron and oxygen ions are evenly distributed (see Fig. 5).

**Fig. 6:**
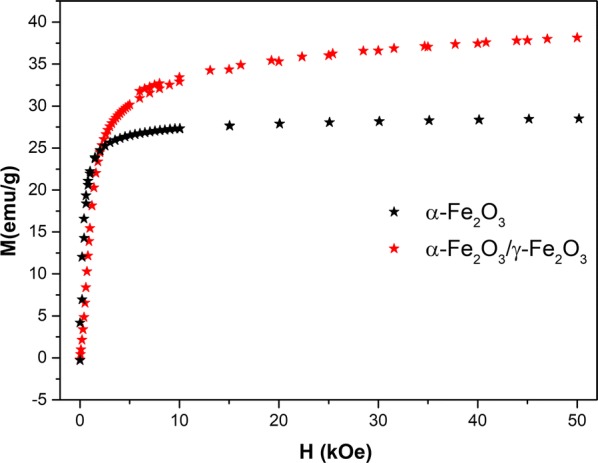
Magnetic field variation of the magnetization in the nanocomposite α-Fe_2_O_3_/γ-Fe_2_O_3_ heated at 300 °C and for comparison α-Fe_2_O_3_ taken at 300 K

In order to further characterize the synthesized IOs nanoparticles, their magnetic properties were determined. The room-temperature magnetic hysteresis measurements of the obtained samples were carried out at 300 K in the applied magnetic field sweeping from 0 to 50 kOe. As shown in Fig. 6, the saturation magnetization (MS) of the nanocomposite α-Fe_2_O_3_/γ-Fe_2_O_3_ was found to be 20 and 35.3 emu g^−1^ at 300 K in comparison with α-Fe_2_O_3_ was to be 5 and 26.6 emu g^−1^. The increase in the saturation magnetization is attributed to the volume increases from hematite (α-Fe_2_O_3_) to maghemite (γ-Fe_2_O_3_). Moreover, the present of γ-Fe_2_O_3_ suggests that the IOs nanoparticles exhibit weak ferromagnetic and soft magnetic behaviours [28] The structure of α-Fe_2_O_3_ can be described as consisting hcp arrays of oxygen ions stacked along the [001] direction. Two-thirds of the sites are filled with Fe^3+^ions which are arranged regularly with two filled sites being followed by one vacant site in the (001) plane thereby forming six fold rings. The structure of γ-Fe_2_O_3_ consists of octahedral and mixed tetrahedral/octahedral layers stacked along [111] direction. All or most of Fe in the trivalent state, and the cation vacancies compensate for the oxidation of Fe^2+^ [29]. The different valence states and cation distribution in the α-Fe_2_O_3_ and γ-Fe_2_O_3_ spinel lattice will cause the change of saturation magnetization and coercivity [28].

### Evaluation of biocompatibility of IOs on RAW 264.7 cells

**Fig. 7:**
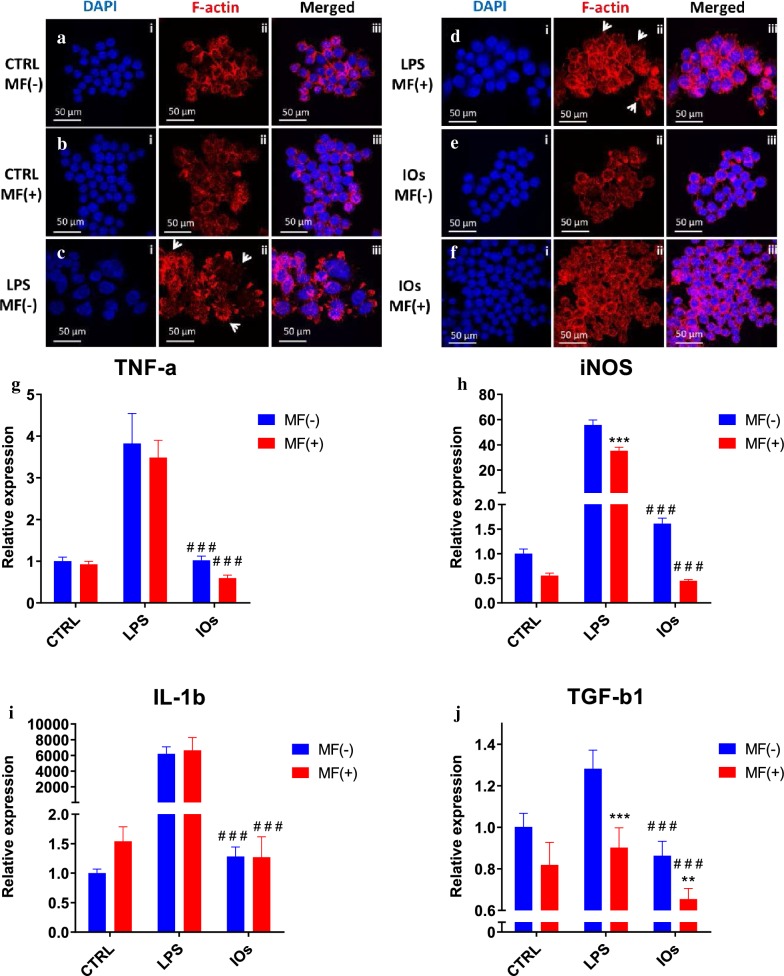
IOs alone and with combination with MF do not elicit immune response. Morphology of macrophages in all investigated groups was investigated using staining for f-actin (**a**–**f**). Activation of macrophages was assessed by qRT-PCR for TNF-a (**g**), iNOS (**h**), IL-1b (**i**) and TGF-b1 (**j**). Results expressed as mean ± SD. Statistical significance indicated as asterisk (*) when comparing the results between corresponding bars representing MF− and MF+ groups, and as number sign (#) when comparing to LPS MF−. **p < 0.01; ###, ***p < 0.001

The effects of manufactured IOs on RAW 264.7 cells are shown at Fig. 7. Cells without LPS treatment displayed spherical shape and their surface was smooth (Fig. 7a, b). On the other hand, cells stimulated with LPS were characterized by spreading and rough surfaces, increased cell size and underwent morphological transformation to dendritic-like cells as they developed robust amount of filopodia (indicated with white arrows). LPS treated cells were used as a positive control as it stimulates M1 polarization which occurs in the presence of inflammatory stimuli and danger signals (Fig. 7c, d). MF alone was not capable to decrease or reverse macrophage activation (Fig. 7d). Interestingly, IOs were shown to be fully biocompatible as no alternation in the morphology of RAW 264.7 was observed either under MF or without exposure (Fig. 7e, f). To further verify the biocompatibility of IOs, we investigated using qRT-PCR, gene expression in RAW 264.7. As expected, TNF-a expression was significantly up-regulated after LPS treatment (Fig. 7g). Addition of IOs without MF exposure did not result in enhancement of TNF-a expression, however IOs combined with MF significantly reduces its mRNA levels in comparison to cells that were not treated with LPS which indicates that IOs are not only biocompatible but also can diminish immune response. In case of iNOS expression, MF alone was able to reduce its expression in LPS treated cells (Fig. 7h). Interestingly, addition of IOs but without MF exposure, resulted in increased iNOS expression in comparison to untreated cells. On the other hand, combination of IOs with MF significantly decreased iNOS expression in comparison to control cells untreated with LPS. mRNA levels of IL-1b were increased in both IOs MF− and IOs MF+ groups (Fig. 7i). Cells treated with LPS after exposure to MF were characterized by decreased TGF-b1 expression (Fig. 7J). In both, IOs MF− and IOs MF+ groups its expression was reduced in comparison to untreated cells.

### Evaluation of inflammation rate in the co-culture of RAW 264.7 with MC3T3 and 4B12

**Fig. 8:**
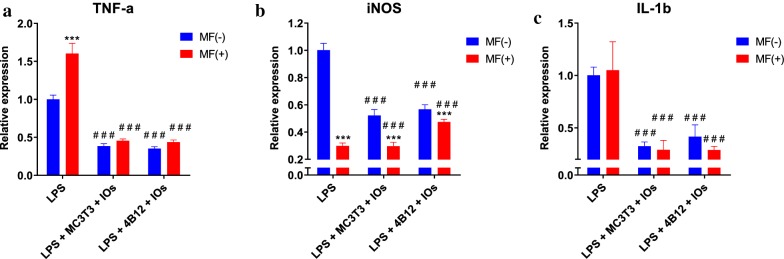
IOs and MF reduce inflammation in co-culture of macrophages with both osteoclasts and osteoblasts. TNF-a (**a**), iNOS (**b**) and IL-1b (**c**) expression was investigated in RAW 264.7 macrophages after co-culture with MC3T3 or 4B12 in the presence and absence of MF. Results expressed as mean ± SD. Statistical significance indicated as asterisk (*) when comparing the results between corresponding bars representing MF− and MF+ groups, and as number sign (#) when comparing to LPS MF−. ###, ***p < 0.001

Expression of TNF-a in both co-cultures was reduced in comparison to LPS treated macrophages (Fig. 8a). Similar phenomenon occurred in the expression of iNOS (Fig. 8b). Interestingly, IL-1 expression was downregulated in all experimental groups (Fig. 8c).

### IOs combined with MF enhance formation of extracellular mineralized matrix in MC3T3 osteoblasts

**Fig. 9:**
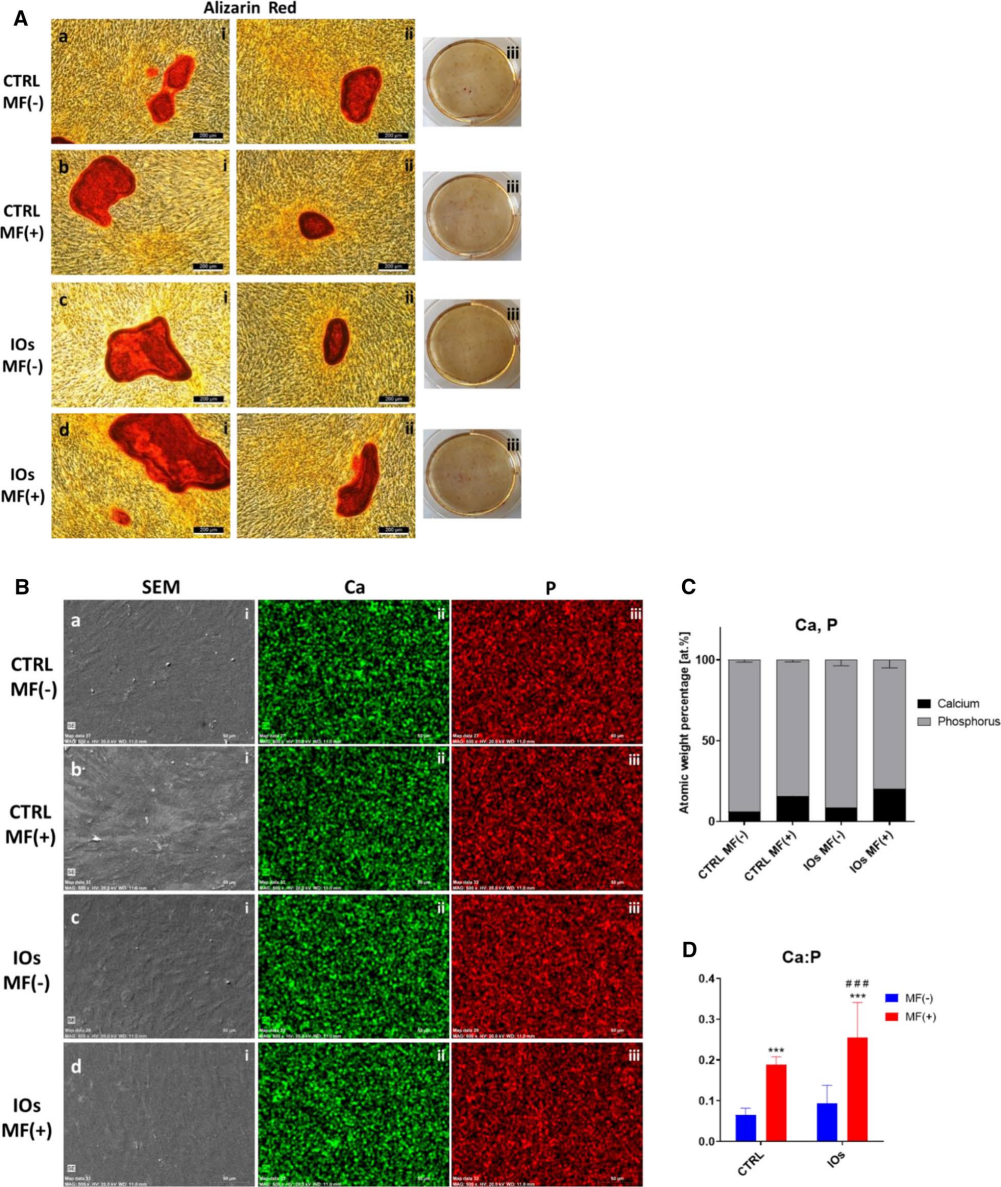
IOs and MF enhance differentiation of MC3T3. Representative photographs from Alizarin Red staining (**A**). Using SEM–EDX morphology of cells as well as C and P distribution was pictured (**B**). Atomic percentage of Ca and P (**C**) and Ca to P ratio (**D**) were calculated. Results expressed as mean ± SD. Statistical significance indicated as asterisk (*) when comparing the results between corresponding bars representing MF− and MF+ groups, and as number sign (#) when comparing to CTRL MF−. ###, ***p < 0.001

After fifth day of IOs supplementation, cells were stained with Alizarin Red in order to observe formation of extracellular matrix (Fig. 9A). Additionally, cells were characterized in SEM microscope with EDX in order to visualize and calculate calcium (Ca) and phosphorus (P) concertation (Fig. 9B). Interestingly, cells treated with IOs, exposed to MF were characterized by increased Ca:P ratio in comparison to remaining groups (Figs. 4d and 9C).

### IOs and MF enhance expression of osteogenic marker genes in MC3T3

**Fig. 10:**
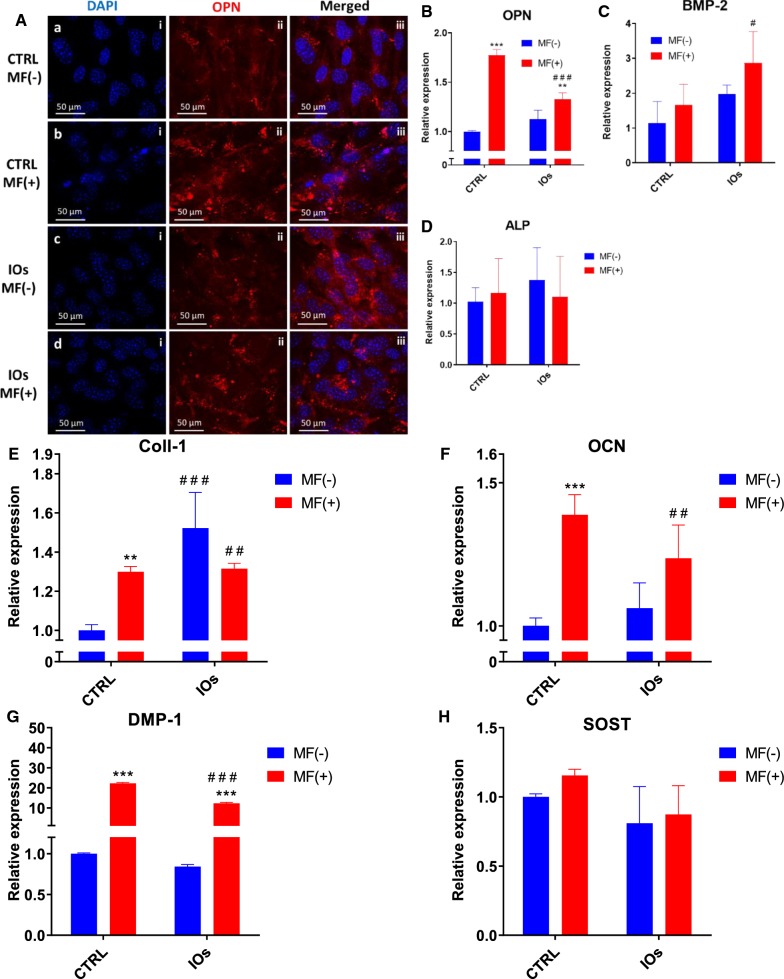
IOs and MF increase MC3T3 differentiation. Representative photographs from OPN staining (**A**) and its relative expression established by qRT-PCR (**B**). Additionally, expression of master regulators of osteogenic differentiation: BMP-2 (**C**), ALP (**D**), Coll-1 (**E**), OCN (**F**), DMP-1 (**G**) and SOST (**H**) was investigated. Results expressed as mean ± SD. Statistical significance indicated as asterisk (*) when comparing the results between corresponding bars representing MF− and MF+ groups, and as number sign (#) when comparing to CTRL MF−. #p < 0.05; ##, **p < 0.01; ###, ***p < 0.001

Immunofluorescence staining for OPN revealed its increased levels in CTRL MF+ and IOs MF+ groups respectively which indicated that MF alone is able to enhance OPN expression (Fig. 10A). These results were confronted with qRT-PCR for OPN as well. Obtained results revealed that MF alone enhance expression of OPN at the higher rate than combination of IOs and MF (Fig. 10B). Contrary, BMP-2 expression was significantly increased in IOs MF+ group (Fig. 10C). No differences were observed in the ALP expression between investigated groups (Fig. 10D). Coll-1 expression was enhanced by MF alone, IOs and the combination of IOs and MF (Fig. 10E). On the other hand, OCN expression was significantly upregulated in CTRL MF+ and IOs MF+ groups when compared to untreated cells (Fig. 10F). The same phenomenon was observed in the expression of DMP-1 (Fig. 10G). No differences between groups were noted in the expression of SOST between groups was observed (Fig. 10H).

### IOs and MF do not affect RUNX-2 and RUNX-1 activity

**Fig. 11:**
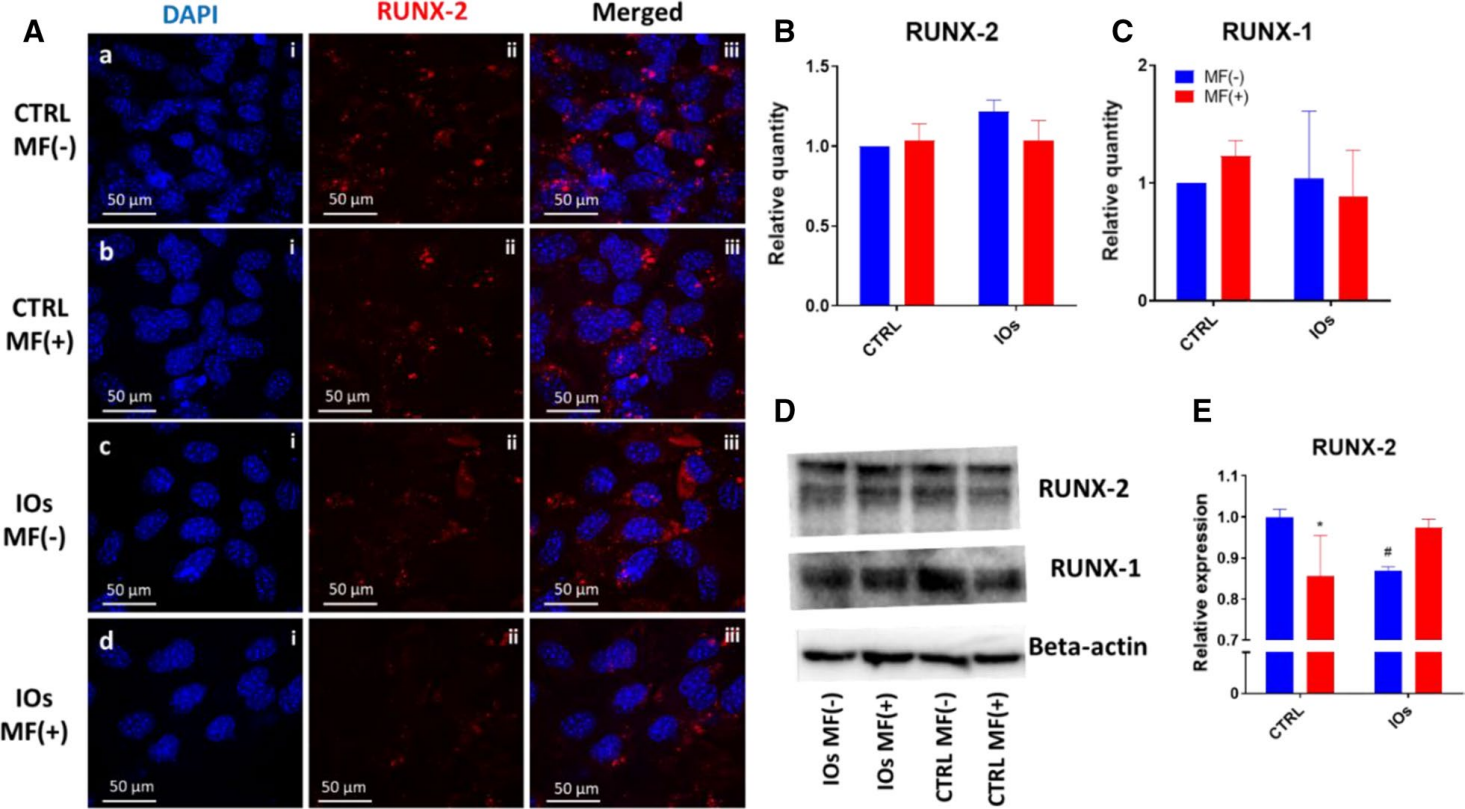
IOs and MF do not affect RUNX-2 and RUNX-1 activity. Representative photographs from RUNX-2 (**A**). Quantification of western blot results for RUNX-2 (**B**) and RUNX-1 (**C**) and representative bands (**D**). qRT-PCR results for RUNX-2 expression (**E**). Results expressed as mean ± SD. Statistical significance indicated as asterisk (*) when comparing the results between corresponding bars representing MF− and MF+ groups, and as number sign (#) when comparing to CTRL MF−. #, *p < 0.05

Immunofluorescence for RUNX-2 (Fig. 11A) showed no significant differences in protein amounts between investigated groups. Similar phenomenon was noted in the amount of RUNX-2 (Fig. 11B) and RUNX-1 (Fig. 11C) as no statistical differences were observed. Representative bars from western blot analysis are shown at Fig. 11D. qRT-PCR results revealed decreased expression of RUNX-2 in IOs MF− group (Fig. 11E).

### IOs and MF affect integrins expression in MC3T3

**Fig. 12:**
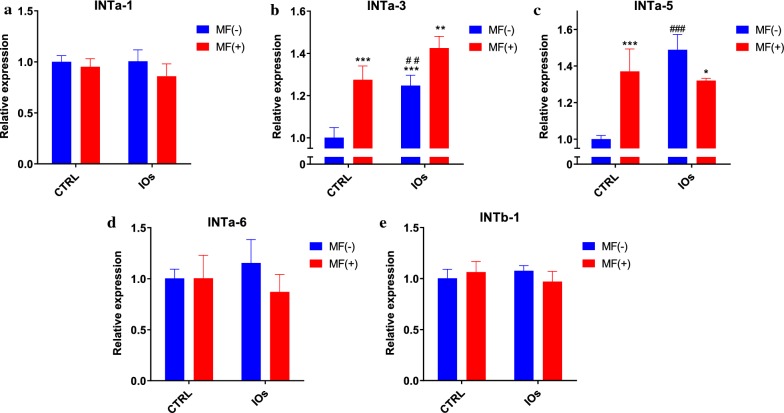
IOs and MF modulate integrin expression in differentiated MC3T3.Exression levels of following integrins were investigated with qRT-PCR: INTa-1 (**a**), INTa-3 (**b**), INTa-5 (**c**), INTa-6 (**d**) and INTb-1 (**e**). Results expressed as mean ± SD. Statistical significance indicated as asterisk (*) when comparing the results between corresponding bars representing MF− and MF+ groups, and as number sign (#) when comparing to CTRL MF−. *p < 0.05, **,##p < 0.01, ***, ###p < 0.001

In order to investigate integrins expression, qRT-PCR was performed after 22th day of differentiation. No differences were observed in the expression of INTa-1 between investigated groups (Fig. 12a). Interestingly, MF significantly enhanced expression of INTa-3 and that effect was further enhanced by the application of IOs (Fig. 12b). Similar results were noticed in the expression of INTa-5 as it was elevated after MF and IOs treatment (Fig. 12c). No statistically significant differences were observed in the mRNA levels of INTa-6 (Fig. 12d) and INTb-1 (Fig. 12e) between investigated groups.

### IOs combined with MF decrease activity of 4B12 osteoclasts

**Fig. 13:**
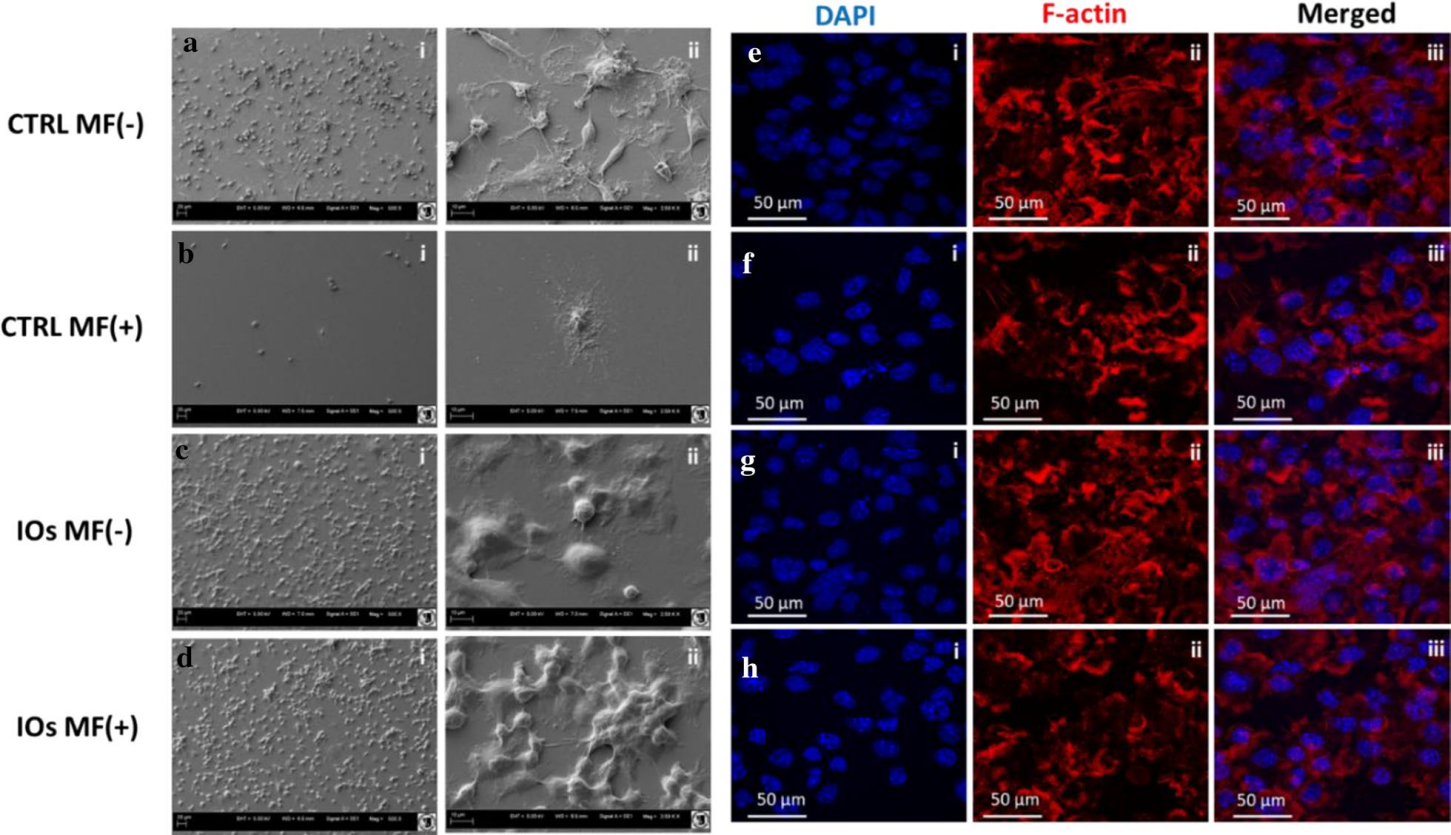
IOs and MF affect morphology of osteoclasts. IOs, MF and their combination affect morphology of osteoclasts. Representative photographs from SEM (**a**–**d**) and f-actin staining (**e**–**h**)

SEM was applied to investigate the morphology of cells cultured in the presence of MF and IOs or in standard condition (Fig. 13a–d). In control group, cells were characterized by typical morphology including formation of filopodia and podosomes extending from the cell membrane, adhering the cell to the surface. Interestingly, treating cells with MF alone, IOs and combination of MF with IOs resulted in profound alternations in their phenotype (Fig. 13b–d). Those cells were more rounded while lacked areas of spreading, membrane ruffles and microvilli. Phalloidin staining allowed for visualisation of F-actin rings which is a feature of functional osteoclasts. Cells from control group displayed well-defined rings on the cell margin (Fig. 13e). The number of signs was slightly reduced in the MF+ group (Fig. 13f). Number of cells with f-actin arranged into ring-like structure at the cell periphery was significantly reduced in both IOs MF− and IOs MF+ groups (Fig. 13g, h). In those groups profound shrinkage of osteoclasts and disruption of actin ring structure was common.

### IOs and MF inhibit osteoclasts activity and affect integrins expression

**Fig. 14:**
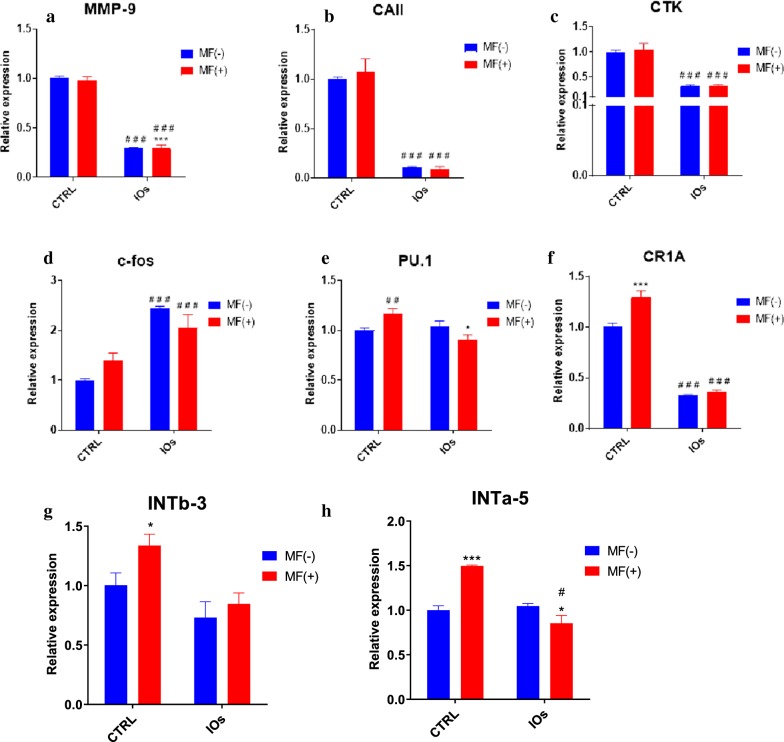
IOs and MF inhibit osteoclasts activity and affect integrins expression. Expression of master regulators of osteoclasts metabolism was investigated with qRT-PCR including: MMP-9 (**a**), CAII (**b**), CTK (**c**), c-fos (**d**), PU.1 (**e**). Additionally, expression of INTb-3 (**g**) and INTa-5 (**h**) was investigated. Results expressed as mean ± SD. Statistical significance indicated as asterisk (*) when comparing the results between corresponding bars representing MF− and MF+ groups, and as number sign (#) when comparing to CTRL MF−. #,*p < 0.05, ##p < 0.01, ***, ###p < 0.001

In order to evaluate the effect of MF and IOs on osteoclasts metabolism, mRNA expression levels of master-regulators of osteoclast activity were examined by RT-qPCR. The results showed that, the expression of MMP-9 (Fig. 14a) and CAII (Fig. 14b) were greatly diminished in IOs MF− and IOs MF+ groups. Similarly, mRNA levels of CTK was decreased after IOs treatment independently of MF exposure (Fig. 14c). Contrary phenomenon was observed in the c-fos expression as its expression was up-regulated in IOs MF− and IOs MF+ groups (Fig. 14d). PU.1 expression was only decreased in IOs MF+ group when compared to control (Fig. 14e), while CR1A expression was diminished in IOs MF− and IOs MF+ groups (Fig. 14f). Obtained data revealed that both, MF and IOs reduced activity of osteoclasts by modulation of master genes related to their metabolism. Furthermore, we decided to investigate the effects of MF and IOs on the expression of INTb-3 and INTa-5 in osteoclasts. Differences in INTb-3 expression was only observed in osteoclast treated with MF only in comparison to untreated cells (Fig. 14g). mRNA levels of INTa-5 were also increased after treating of cells with MF only while decreased in IOs MF+ group in comparison to control cells (Fig. 14h).

### MF and IOs downregulate activity of master osteoclast protein

**Fig. 15:**
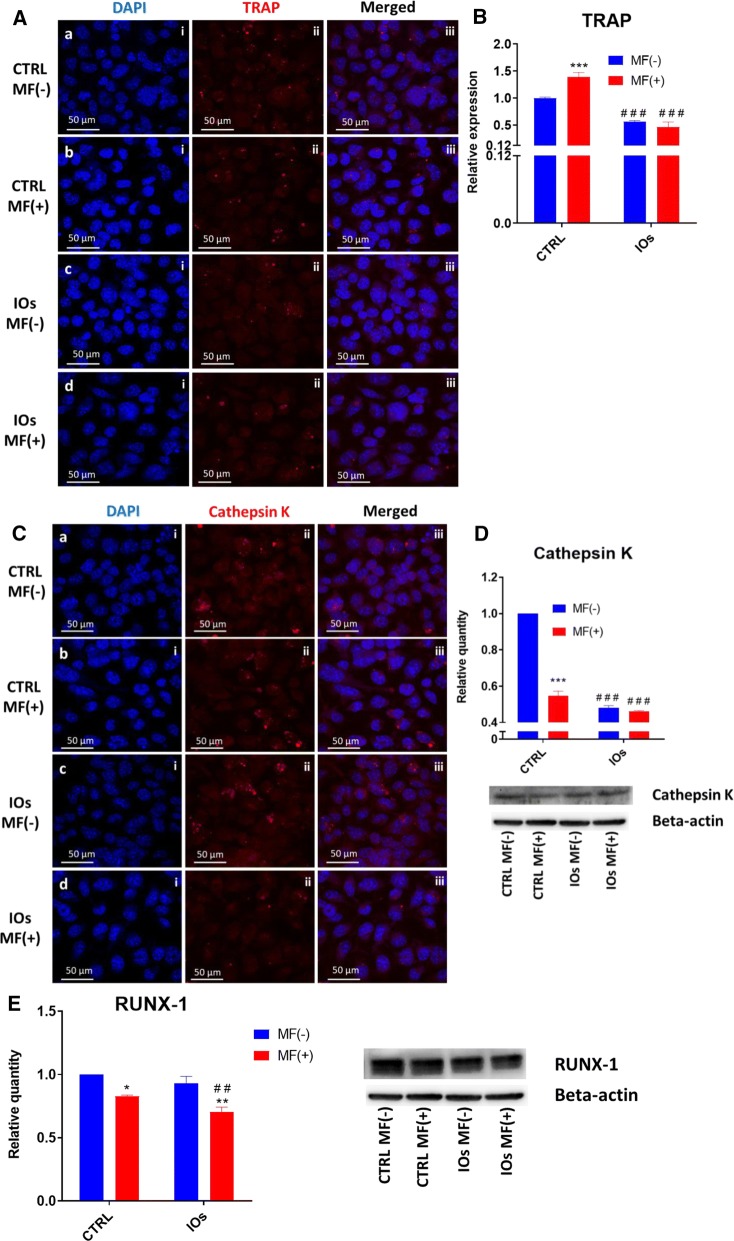
IOs and MF modulate RUNX-1 and RANKL in osteoclasts. Representative photographs from immunofluorescence TRAP staining (**A**) supported by qRT-PCR (**B**) data. Additionally, immunofluorescence staining for Cathepsin K (**D**) was performed and support with western blot analysis (**E**). As revealed by western blots, IOs and MF reduce amount of RUNX-1. Results expressed as mean ± SD. Statistical significance indicated as asterisk (*) when comparing the results between corresponding bars representing MF− and MF+ groups, and as number sign (#) when comparing to CTRL MF−. *p < 0.05, ##, **p < 0.01, ***, ###p < 0.001

In order to evaluate how IOs and MF affect osteoclasts we measured activity of two major regulators of osteoclasts metabolism- tartrate-resistant acid phosphatase (TRAP) and Cathepsin K. Immunofluorescence staining for TRAP revealed its increased levels in untreated cells and those exposed to MF only (Fig. 15A). The amount of TRAP was comparable between IOs MF− and IOs MF+ groups. Furthermore, we examined mRNA levels of TRAP in cells using qRT-PCR (Fig. 15B). Obtained results indicated on its increased expression in untreated cells and those exposed to MF only while decreased expression was observed in IOs MF− and IOs MF+ groups which correlates with immunofluorescence data. Similar results were obtained from Cathepsin K immunofluorescence. Obtained data indicated that its levels decreased after treating cells with IOs and MF (Fig. 15C). Furthermore, these observations were supported by western blot data (Fig. 15D). Western blots also revealed, that MF alone and in combination with IOs reduce amount of runt-related transcription factor 1 (RUNX-1) protein in osteoclasts (Fig. 15E).

### MF and IOs increase apoptosis of osteoclasts

**Fig. 16:**
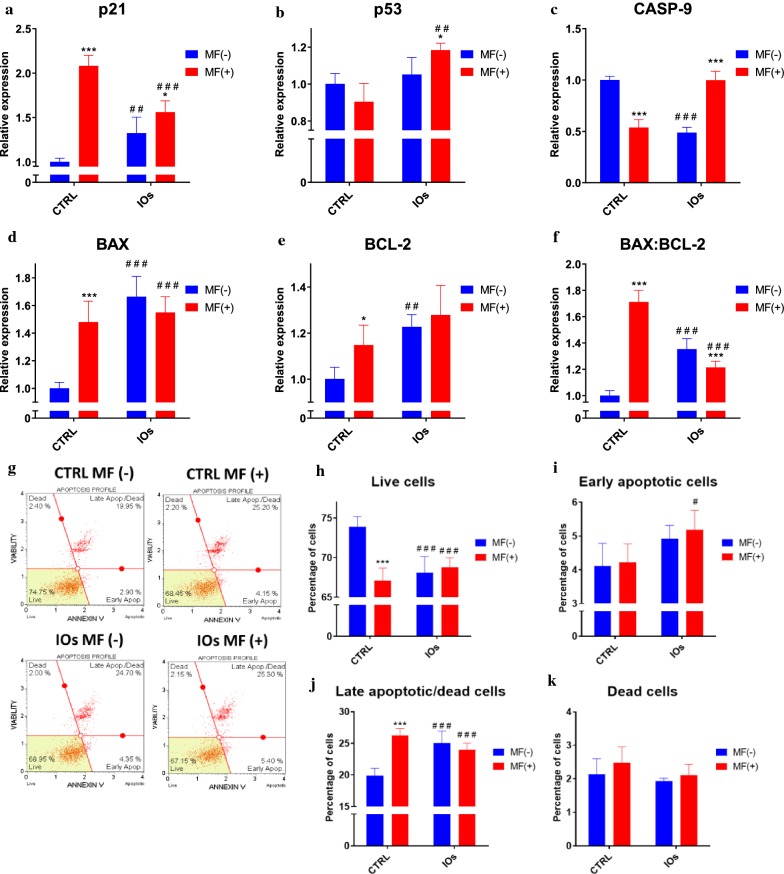
IOs and MF induce apoptosis in osteoclasts. Expression of apoptosis related genes including p21 (**a**), p53 (**b**), CASP-9 (**c**), BAX (**d**) and BCL-2 (**e**). Furthermore, based on the relative gene expression the ratio of BAX:BCL-2 was calculated (**f**). Representative graphs from Muse^®^ Annexin V and Dead Cell Assay Kit (**g**). Based on the obtained data, percentage of live cells (**h**), early apoptotic cells (**i**), late apoptotic/dead cells (**j**) and dead cells (**k**) was calculated. Results expressed as mean ± SD. Statistical significance indicated as asterisk (*) when comparing the results between corresponding bars representing MF− and MF+ groups, and as number sign (#) when comparing to CTRL MF−. #,*p < 0.05, ##, **p < 0.01, ***, ###p < 0.001

**Fig. 17:**
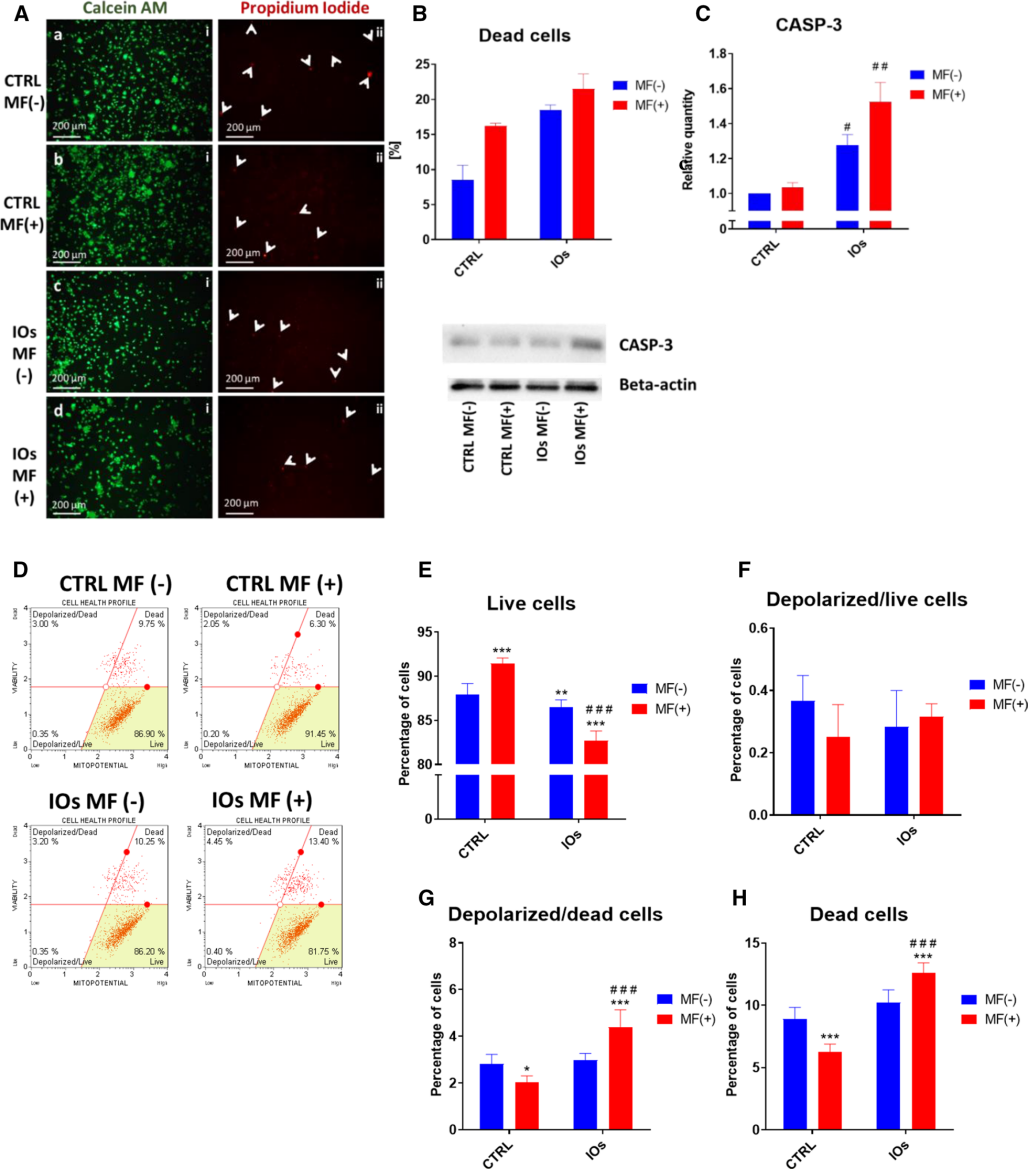
IOs and MF affect mitochondrial membrane potential and contributes to enhance apoptosis in osteoclasts. Representative photographs from Calcein A.M. and Propidium iodide (**A**) and their quantification (**B**) revealed increased number of apoptotic cells in IOs and MF groups. Western blot analysis revealed increased amount of CASP-3 in IOs MF− and IOs MF+ groups. To investigate mitochondrial condition in cells Muse^®^ MitoPotential analysis was performed (**D**). Based on the obtained data, percentage of live cells (**E**), depolarized/live cells (**F**), depolarized/dead cells (**G**) and dead cells (**H**) was calculated. Results expressed as mean ± SD. Statistical significance indicated as asterisk (*) when comparing the results between corresponding bars representing MF− and MF+ groups, and as number sign (#) when comparing to CTRL MF−. #,*p < 0.05, ##, **p < 0.01, ***, ###p < 0.001

In order to investigate whether MF and IOs can trigger apoptosis in osteoclasts, we tested expression of genes related to apoptotic pathway. Interestingly, expression of cyclin-dependent kinase inhibitor 1 (p21) was up-regulated the most in MF only group (Fig. 16a), however IOs and their combination with MF also increased p21 mRNA levels in comparison to untreated cells. On the other hand, p53 tumour suppressor (p53) expression was only affected by combination of MF with IOs (Fig. 16b). Similar phenomenon was observed in the expression of caspase 9 (CASP-9) (Fig. 16c). Upregulation of bcl-2-like protein 4 (BAX) expression in comparison to control group was observed in each of investigated groups (Fig. 16d), similarly to B cell lymphoma 2 (BCL-2) (Fig. 16e). Ratio of BAX:BCL-2 indicated on increased apoptosis in all of experimental groups (Fig. 16f). Obtained data indicates that MF and IOs activate apoptosis in osteoclasts. To further investigate the apoptosis in cells, we performed analysis with Muse Cell Analyser and Muse^®^ Annexin V and Dead Cell Assay Kit. Representative plots are shown in Fig. 16g. Data quantification revealed decreased number of viable cells in all experimental groups (Fig. 16h). Number of early apoptotic cells was increased in IOs MF+ (Fig. 16i), while late apoptotic cells in all experimental groups (Fig. 16j). However, the number of dead cells does not differ significantly between groups (Fig. 16k). Thus, we decided to further evaluate apoptosis and revealed whether it is related to mitochondrial dysfunction. Fluorescence staining for viable and dead cells with Calcein A.M./Propidium Iodide (Fig. 17A) and its quantification (Fig. 17B) revealed increased number of dead cells in all of the experimental groups. Western blots results indicted on increased amount of caspase 3 (CASP-3) in IOs MF− and IOs MF+ (Fig. 17C). To investigate mitochondrial condition in cells we performed Muse^®^ MitoPotential analysis and obtained representative plots are shown in Fig. 17D. Obtained data indicated decreased number of viable cells in IOs MF− and IOs MF+ (Fig. 17E), while no differences regarding number of live, depolarized cells were observed (Fig. 17F). On the other hand, increased number of depolarized and dead cells was noted in IOs MF+ (Fig. 17G, H). Obtained results revealed that cells undergo apoptosis which correlates with mitochondrial dysfunction.

## Discussion

Bone fractures especially in osteoporotic patients require advanced therapeutic techniques which are able to modulate the metabolism of recipient cells. As during osteoporosis imbalance between activity of bone forming and bone resorbing cells contributes to the development of disease and impairs tissue homeostasis, novel therapeutic approaches should focus on the application of agents able to restore cellular functions. The stimulation of osteoblasts for differentiation and inhibition of osteoclasts activity seems to be a key component in the course of osteoporotic bone regeneration. Recently, we have shown that magnetic iron nanoparticles (IOs) enhance progenitor cells osteogenic activity which supports their application in regenerative medicine [19].

As magnetic field was shown to enhance osteogenic differentiation, augment bone and wound healing [30, 31], we decided to fabricate α-Fe_2_O_3_ and γ-Fe_2_O_3_ IOs and investigate their effect alone and in combination with MF on osteoblasts, osteoclasts and macrophages in order to evaluate their therapeutic potential. We show that IOs induce osteoblasts differentiation and inhibits osteoclasts activity while being completely biocompatible. What is more, these effects can be strengthen up when MF is applied to the cell culture environment.

Fabricated IOs crystallite size was estimated to be 37 nm for α-Fe_2_O_3_ and 88 nm for γ-Fe_2_O_3_. We also investigated particles morphology with SEM technique and revealed that IOs are characterized by relatively homogeneous distribution and show tendency to aggregate into spherical-like objects. While, the typical hydrodynamic size of the studied IOs was in the range 190–220 nm. It needs to be pointed out that uptake of nanoparticles by cells mostly is affected by the surface charge of the particles. Their surface charge can extremely influence on the biological system response including absorption, distribution, metabolism, and excretion upon exposure. In many cases cell membranes possess large negatively charged surface which is unfavorable for particles with negative zeta potential distribution. In case of the studied IOs, the zeta potential exhibits negative value equal − 21.50 mV. However, there are some reports that negatively charged particles can easily penetrate through the cell membrane due to the possible presence of positively charged domains [32]. Another solution to improve the cell-particle interaction may be to use surface modification of nanoparticles with specific ligands, which then ultimately increase the surface charge. Nevertheless, the possible cellular uptake mechanisms of negatively charged nanoparticles needs further study. Especially, it is important applying more appropriate environment (similar to physiological condition) than aqueous solution, as well as using model molecules widely utilized in biomedical experiments (e.g. bovine serum albumin and lysozyme [33] which in turn enable a detailed insight into the protein-nanoparticle interaction.

Here, we investigated the defect of IOs under MF condition on differentiated osteoblast and osteoclast as well as on macrophages polarization. MF was applied to cells using lab developed device in which culture plates were exposed to 0.2 T. In first stage of the experiment, cytobiocompability of fabricated IOs was investigated with RAW 264.7 cells where cells treated with LPS served as a control. We have shown that neither IOs or MF can activate immune response. What is more, further experiments revealed, that application of IOs and MF diminish macrophages polarisation towards M1 phenotype in co-culture with both, osteoclasts and osteoblasts. Similar results were obtained by Beck-Speier et al. [34] who revealed that Fe_2_O_3_ particles enhanced expression prostaglandin E2 (PGE2) as anti-inflammatory marker and decreased pro-inflammatory interleukin-6 expression in rat alveolar macrophages. That mechanism is probably dose dependent as it was shown that those IOs can induce liver and lung tissue inflammation while administered in high dosage [35]. Importantly, here we have found that IOs but only in combination with MF can significantly diminish expression of TNF-a, a cytokine which contributes to osteoporosis by triggering RANKL-induced osteoclast formation [36].

In the next step, efforts have been made to explore if and how IOs enhance osteogenesis of MC3T3 cells. We have found that IOs combined with MF increased Ca:P ratio, enhanced expression of OPN, Coll-1, OCN, DMP-1 and BMP-2- crucial markers for osteogenesis. Pro-osteogenic properties of IOs were also proved by Xia et al. [37] who revealed that calcium phosphate scaffold doped with iron oxide nanoparticles enhanced osteogenesis in dental pulp stem cells. Similar results were found by Wang et al. [38] who discovered that magnetic iron oxide nanoparticles (IONPs) enhance osteogenesis in mesenchymal stem cells (MSCs) via upregulation of long noncoding RNA INZEB2. IOs enriched with poly-l-lysine are internalised by cells and exerts wide range of metabolic alternations. Second, when MF is applied mechanical stress signals between IOs and cell membrane promotes osteogenesis [39]. Furthermore, if IOs after internalisation are transferred to lysosomes, free iron is released. It is especially important due to the fact that iron is an essential element for normal bone metabolism and its deficiency leads to osteoporosis. Fe(III) for example, participate in many biological aspects of cells being part of proteins and enzymes. It was shown that MF with different strengths modulates iron content in cells and thus contributes to enhanced osteoblasts differentiation [40]. What is worth noting, IOs and MF elevated the expression of OPN which is a crucial regulator of bone formation under mechanical stress. Finally we observed significantly enhanced expression of DMP-1 a protein which controls osteocyte maturation. We also observed upregulation of Coll-1 which expression is diminished in MSC isolated from osteoporotic patients [41]. It is tempting to speculate, that restoration of proper Coll-1 synthesis may help to restore bone homeostasis and stop disease progression. Interestingly, MF and IOs alone decreased amount of RUNX-2 which rather supports their combined application in order to enhance osteogenic differentiation. Observed effect might be explained by the action of MF and IOs, since it we showed in our previous research that MF promotes osteogenic differentiation potential in stem progenitor cells through shedding of extracellular macrovesicle re (EXMV’s) rich in growth factors involved in osteogenesis regulation [42, 43].

Integrins belong to the receptors for extracellular matrix (ECM) proteins and play a crucial role in cell migration, adhesion, proliferation and survival. Here we have found that IOs and MF significantly upregulated the expression of INTa-3 and INTa-5. Interestingly, INTa-3 is involved in fibronectin deposition into pericellular matrix [44] while INTa-5 was shown to promote the osteogenic differentiation of human periodontal ligament stem cells [45]. Also it was shown that INTa-5 expression increase during osteogenesis progression [46]. We speculated that, enhanced expression of INTa-3 and INTa-5 is triggered by IOs and MF and in consequence activate intracellular signal network which leads to augment of osteogenic differentiation. Thus modulation of INTa-3 and INTa-5 may become a therapeutic strategy to promote efficient bone regeneration [47].

Pathophysiology of osteoporosis results from the imbalance between bone formation and resorption process leading to decreased bone density and susceptibility to fractures. Thus it is especially important to not only enhance activity of osteoblasts but at the same time reduce osteoclasts action. Cathepsin K is secreted by activated osteoclasts during bone resorption and is crucial for the degradation of bone matrix protein, especially Coll-1 [48]. Herein we have found that MF and IOs significantly diminished synthesis of that protein by osteoclasts. Interestingly, current therapeutic strategies which belongs to cathepsin K inhibitors do not exert satisfactory results [49]. Similar phenomenon was observed in the expression of TRAP which participate in bone and OPN degradation [50]. Our results revealed that IOs alone and in combination with MF significantly decrease TRAP levels in osteoclast. Simultaneously, we observed downregulation of other osteoclasts marker gene expression e.g. MMP-9, CAII, CTK and CR1A. For our knowledge this is a first report showing that Fe_2_O_3_ nanoparticles are able to reduce osteoclasts activity. Research performed by Lee et al. [51] revealed that similar to Fe_2_O_3_, Fe_3_O_4_ nanoparticles doped with aldosterone, are able to control osteoporosis progression by destroying osteoclasts through thermolysis. Contrary to our results, it was also shown that iron can stimulate osteoclasts differentiation via NF-κB signalling pathway [52] and through increase of RANKL/reactive oxygen species levels [53]. Inhibition of RANKL and other master regulators of osteoclastogenesis by IOs might be osteoprotective mechanism against excessive osteoclasts activity observed during osteoporosis. In next step we attempted to evaluate the mechanisms by which IOs decrease osteoclasts activity. Obtained data revealed that IOs promoted apoptosis through triggering apoptotic signalling pathways. IOs induced expression of BAX, p21, Casp-9 and decreased mitochondrial membrane potential which indicates on the activation of mitochondrial apoptosis pathway. Simultaneously activation of p53 and Casp-3 suggest the activation of intrinsic pathway as well. For our knowledge it is a first report showing that IOs can induce apoptosis specifically in osteoclasts while enhanced osteogenic properties of osteoblasts. Similar research has only been performed by Lee et al. [51] who fabricated magnetic nanoparticles however with Fe_3_O_4_ combined with alendronate for the treatment of osteoporosis. They have found that, fabricated nanoparticles decreased osteoclasts cell survival rate and be applied as a stable MRI contrast agent.

## Conclusions

Osteoporosis prevalence is rapidly growing. It is estimated that costs for osteoporosis-related fractures will increase with 50% from 2010 to 2030 [54]. The strategies to overcome the disease aim to increase body mass and limit occurrence of fractures. For the first goal, only one drug is approved by FDA- intermittent parathyroid hormone (PTH), however at the same time it can also enhance bone resorption. Bone resorption is limited with the application of anti-resorptive agents, such as nitrogen containing bisphosphonates (alendronate, risedronate) and denosumab [55] although they were shown to exerts effects on osteoblast and osteocytes too [56]. Development of new drugs should engage both mechanisms- modulate activity of osteoclasts and osteoblasts in order to restore tissue homeostasis. In this paper we have shown potential utility of IOs nanoparticles in the treatment of osteoporosis as fabricated nanoparticles inhibited osteoclasts activity while enhanced osteogenic differentiation of osteoblast. Obtained results indicate that application fabricated IOs might become a therapeutic agent for the treatment of bone disorders related to bone loss, including osteoporosis. Yet mechanism behind this phenomenon needs to be further elucidated especially in vivo model in order to fully explore IOs therapeutic potential.

## Methods

### All of the reagents used in the study were purchased from Sigma-Aldrich (USA), unless indicated otherwise

#### IOs synthesis

The iron oxide nanoparticles were synthesized by a modified sol–gel method. Iron (III) nitrate nonahydrate, Fe(NO_3_)_3_‧9 H_2_O, (98+ %, Alfa Aesar) as a precursor was gelled by using ethylene glycol (ultrapure, Avantor Performance Materials Poland S.A.) in a molar ratio of 1:10. Firstly, the solution was stirring for 2 h at 40 °C and then was heated under vigorous stirring until gel formation. The nanocrystalline iron oxides powder were obtained after gel calcination under air atmosphere at 300 °C.

#### Characterisation of fabricated IOs

The formation of an iron oxide structure was confirmed by using X-ray powder diffraction (XRPD) technique in a 2θ range of 15–80° with X’Pert Pro PANalytical X-ray diffractometer (Cu Kα1: λ = 1.54060 Å). The experimental XRPD patterns were compared with the standards from Inorganic Crystal Structure Database (ICSD) and the mean size of crystallites was calculated using Rietveld refinement. Raman measurements were carried out with a Micro-Raman system Renishaw inVia equipped with a Leica DM 2500 M microscope and a CCD camera as detector. The microstructure, morphology and elemental mapping of iron oxide nanoparticles were investigated by using FEI Nova NanoSEM 230 scanning electron microscopy equipped with an EDS spectrometer (EDAX GenesisXM4) and operating at an acceleration voltage in the range 3.0–15.0 kV and spot 2.5–3.0.

Magnetic measurements were performed using a Quantum Design Physical Property Measurement System (PPMS) with vibrating sample magnetometer (VSM) option at temperatures between 2 and 380 K in 100 and 1000 Oe applied magnetic field (MF). Nanocrystalline powders were compacted into disc-shaped samples and further crashed into small pieces to avoid displacements under experimental conditions.

Mӧssbauer spectroscopy (MS) was used to identify the relative fractions of the iron oxide phases. The room-temperature ^57^Fe Mössbauer spectrum was recorded in transmission geometry with a conventional constant-acceleration spectrometer, using a ^57^Co-in-Rh standard source with a full width at half maximum (FWHM) of 0.24 mm/s. The obtained MS spectrum was analysed using a least-squares fitting procedure which allows to determine parameters such as isomer shift (IS), quadrupole splitting/shift (QS), hyperfine magnetic field (B), relative spectral areas (C) and spectral linewidths (Γ) which are related to different chemical states of the Mössbauer probes. All the IS values presented in this paper are related to the α-Fe standard at room temperature.

Zeta potential and hydrodynamic size of the iron oxide suspension were determined by Phase Analysis Light Scattering (PALS) and Dynamic Light Scattering (DLS) with a Zetasizer Nano ZS apparatus from Malvern Instruments operating under He–Ne 633 nm laser and equipped with the Dispersion Technology Software for data collection and data analysis. The starting concentration of nanoparticles suspension was around 500 μg mL^−1^ and was further diluted with de-ionized water to eliminate errors connected with too high or too low amount of analyzed object. Each measurement was repeated three times with fixed concentrations of particles to achieve reliable statistics.

#### Cell culture

Undifferentiated MC3T3 were cultured in MEM-alpha (Gibco, A10490-01) supplemented with 10% fetal bovine serum (FBS). Prior differentiation medium was changed for MEM-alpha with 10% FBS supplemented with 50 ug/mL l-ascorbic acid (A5960) and 10 mM B-glycerophosphate disodium salt hydrate (G9422). Culture media for undifferentiated 4B12 consisted of MEM-alpha (Gibco, 12561-056), 30% calvaria-derived stromal cell conditioned media (CSCM) and 10% FBS [57]. In order to induce differentiation of 4B12 into functional osteoclasts, they were maintained in culture media (as described above) supplemented with 1.5 ng/mL M-CSF form mouse (Sigma Aldrich, SRP3221) and 1.5 ng/mL Recombinant Mouse TRANCE/RANK L/TNFSF11 (R&D Systems, 462-TEC). RAW 264.7 were cultured in culture medium consisted of DMEM with 4500 mg/L glucose supplemented with 10% fetal bovine serum (FBS). Medium was refreshed every 2–3 days. The cells were passaged when grown to 80% confluence using recombinant cell-dissociation enzyme StableCell Trypsin.

#### Preparation of IOs for in vitro experiments

In order to perform the experiments IOs were sonicated in ultrasonic bath for 1 h. Next, they were diluted 1:1 in DMEM/F12 medium and poly-l-lysine (10% v/v) was added to the solution. Following 2 h incubation on plate roller, solution was centrifuged at 12,000×*g* for 10 min. Supernatant were discarded, remaining IOs were re-suspended in DMEM/F12 at the initial volume and filtered through 0.22 µm syringe filter. IOs were added to the cells at the concentration of 68.7 ug/mL.

#### Experimental setting

Cells were exposed to magnetic field using system was designed at the Institute of Low Temperature and Structure Research Polish Academy of Science in Wroclaw as described previously [19]. The MF strength equalled 0.2 T. The cell culture plate of following size 127.89 × 85.6 × 19.69 mm was placed between magnets, and all systems were installed in CO_2_ incubator.

Undifferentiated MC3T3 cells were seeded onto 24-well plates and maintained in MEM-alpha (Gibco, A10490-01) with 10% FBS. When cells reached 80% confluence culture media was exchanged for MEM-alpha with 10% FBS supplemented with 50 ug/mL l-ascorbic acid (A5960) and 10 mM B-glycerophosphate disodium salt hydrate (G9422) in order to induce differentiation. At 18th day of differentiation process IOs were added to the medium and cells were exposed to MF for 15 min daily for 5 days.

Undifferentiated 4B12 cells were seeded onto 24-well plates and maintained in differentiation medium- MEM-alpha (Gibco, 12561-056), 30% CSCM, 10% FBS, 0.1% RANKL and 0.01% M-CSF. At 11th day of differentiation, IOs were added to the medium and cells were exposed to MF for 15 min daily for 5 days.

RAW 264.7 at a density of 5 × 10^5^ cells/mL were seeded onto wells of a 24-well plate. Next, lipopolysaccharide (LPS, 1 μg/mL) and IOs were added to the culture media for another 6 h. After 6 h LPS were removed from the culture medium while IOs remained. The same and following day cells were exposed to MF for 15 min.

#### Alizarin Red staining

After 22th day of differentiation in culture and experimental conditions, MC3T3 osteoblasts were with Alizarin Red in order to visualize extracellular matrix mineralisation. Prior observations, cells were fixed with 4% paraformaldehyde (PFA) at room temperature for 15 min. Next specimens were washed with phosphate buffer saline (PBS) three times. 10% solution of Alizarin Red was applied to cells for 10 min followed by PBS wash. Samples were observed under an inverted microscope (Leica DMI1).

#### Visualization of cellular morphology

Detailed morphology of investigated cells was investigated using scanning electron microscopy (SEM, EVO LS15, Zeiss). For MC3T3 pictures were obtained after 22th day of differentiation while for 4B12 15th day of differentiation. Procedure of samples preparation was performed as described previously [58]. Briefly, after fixation in 4% PFA, specimens were dehydrated in graded ethanol series, sprinkled with gold and (ScanCoat 6, UK) and analysed with SE1 detector at 1 kV of filament tension. Calcium and phosphorus concentration was assessed with SEM with energy dispersive X-ray analysis (SEM/EDX). The quantax detector (Brüker) with 10 kV of filament tension was applied to perform a line scan analysis of randomly selected cells. The obtained values were presented as weight percentage (wt %).

F-actin was stained in cells with Phalloidin Atto 590 solution. Prior staining, cells were fixed with 4% PFA and their membranes were permeabilized with 0.2% Tween 20 in PBS for 15 min. Then phalloidin solution (1:800) was added to cells for 40 min. Nuclei were counterstained with 4′,6-diamidino-2-phenylindole (DAPI). Cells were observed under confocal microscope (Leica TCS SPE). Staining was performed after 15th day of experiment for 4B12 and after day 2nd for RAW 264.7.

Viable and dead cells were stained with Calcein A.M (3 μM) and propidium iodide (2.5 μM) respectively. Cells were incubated with dyes for 30 min at 37 °C. The cells were captured using an epifluorescence microscope (Axio Observer A.1) and images were taken using a PowerShot camera (Canon).

#### Assessment of apoptosis rate

The mitochondrial membrane potential and was assessed with Muse^®^ MitoPotential kit (Merck) while apoptosis and necrosis were estimated with Muse^®^ Annexin V and Dead Cell Assay Kit (Merck). Analyses were performed in accordance to manufacturer’ instructions using Muse™ Cell Analyzer. The analysis was performed in triplicates.

#### Immunofluorescence

Prior experiments, cells were fixed with 4% PFA, washed with PBS and permeabilized with 0.2% Tween 20 in PBS for 15 min. After additional washing, cells were incubated with proper antibodies and 10% goat serum at 4 °C overnight. Following antibodies and their dilution were applied Col-1A1 1:50, RUNX-2 1:50, Cathepsin K 1:50, TRAP 1:50, and osteopontin (OPN) 1:1000 (supplier and catalogue numbers are shown in Table 2). Atto-590-conjugated secondary antibodies were applied for 1 h to detect the signal. Nuclei were conterstained with DAPI. Cells were observed and imaged using confocal microscope (LEICA TSC SPE) and analysed with Image J software [59].

**Table 2:** List of antibodies used in the experiments

Protein	Manufacturer, catalog no.	Dilution
RUNX-1	Santa Cruz Biotechnology, INC. sc-365644	1:100
RUNX-2	Santa Cruz Biotechnology, INC. sc-390351	1:100
Cathepsin K	Santa Cruz Biotechnology, INC. sc-48353	1:100
TRAP	Santa Cruz Biotechnology, INC. sc-376875	1:100
OPG	Santa Cruz Biotechnology, INC. sc-390518	1:50
RANKL	Santa Cruz Biotechnology, INC. sc-377079	1:100
OPN	Abcam, ab8448	1:1000
COL1-A1	Santa Cruz Biotechnology, INC. sc-293182	1:50
CASP-3	Invitrogen, 43-7800	1:250
Beta-actin	Sigma Aldrich, A5441	1:10000
Anti-mouse IgG, HRP conjugated	Sigma Aldrich, A3562	1:5000
Anti-rabbit IgG, HRP conjugated	Sigma Aldrich, A3687	1:5000

*RUNX-1* Runt-related transcription factor 1, *RUNX-2* runt-related transcription factor 2, *TRAP* tartrate-resistant acid phosphatase, *OPG* osteoprogerin, *RANKL* receptor activator for nuclear factor κ B ligand, *OPN* osteopontin, *COL1-A1* collagen type I alpha 1 chain, *CASP-3* caspase 3, *HRP* horseradish peroxidase

### Western blotting

After last day of the experiment (22th day for MC3T3, 15th for 4B12 and 2nd for RAW 264.7), cells were detached from the culture flasks and homogenised with RIPA buffer containing protease inhibitor cocktail. Next samples were centrifuged (20 min at 14,000×*g*, 4 °C) and supernatants were stored at − 80 °C before analysis. Protein amount in each sample was estimated with Pierce™ BCA Protein Assay Kit (Life Technologies, USA). Samples were subjected to SDS–polyacrylamide gel electrophoresis at 100 V for 90 min using Mini-PROTEAN Tetra Vertical Electrophoresis Cell (Bio-Rad, USA). Protein were transferred onto polyvinylidene difluoride (PVDF) membranes (Bio-Rad, USA) using a Mini Trans-Blot ^®^ Cell (Bio-Rad, USA) at 100 V for 1 h at 4 °C. Blocking was performed by incubation of membranes in 5% non-fat milk in TBST for 2 h. Selected proteins were detected by overnight incubation with primary antibodies. Next, membranes were incubated with secondary HRP-conjugated antibodies (dilution 1:5000 in TBST for 2 h). Antibodies and their dilutions are shown in Table 2. Chemiluminescent signals were detected using Chemiluminescent/Fluorescent Substrate Kit (Vector Laboratories, Inc. SK-6604) with ChemiDoc MP Imaging System (Bio-Rad, USA) and quantified with Image Lab Software (Bio-Rad, USA).

#### Analysis of gene expression using quantitative real time polymerase chain reaction (qRT-PCR)

Total RNA was extracted from cells using phenol–chloroform method, as previously described by Chomczynski and Sacchi [60]. The quantity and quality of RNA was estimated using a spectrophotometer (Epoch, Biotek). 150 ng of RNA was used for cDNA synthesis using RevertAidFirst Strand cDNA Synthesis Kit (Thermo Fisher Scientific, USA) following by gDNA digestion with DNase I RNase-free Kit (Thermo Fisher Scientific, USA). qRT-PCR was performed as described previously [61] using CFX ConnectTM Real‐Time PCR Detection System (Bio‐Rad). Using RT-PCR, we have investigated the expression of genes involved in the regulation of osteogenic differentiation, osteoclasts activity, apoptosis and inflammation. The average fold change in the gene expression was calculated by the 2 ^−ΔΔCT^ method using GAPDH as the housekeeping gene [62]. Sequences of the primers are attached in the Additional file 1.

### Statistics

All experiments were performed at least in three replicates. Differences between experimental groups was estimated using the one‐way ANOVA with Tukey’s test. Statistical analysis was conducted with GraphPad Prism Software (La Jolla, CA, USA). Differences with probability of P < 0.05 were considered significant. Statistical significance indicated as asterisk (*) when comparing the between corresponding MF− and MF+ groups, and as number sign (#) when comparing to CTRL MF−.

## Supplementary information

**Additional file 1.** The list of the primers.

## Data Availability

The datasets used and/or analysed during the current study are available from the corresponding author on reasonable request.

## References

[1] Demontiero O, Vidal C, Duque G (2012). Aging and bone loss: new insights for the clinician. Ther Adv Musculoskelet Dis..

[2] World Health Organization (1994). Assessment of fracture risk and its application to screening for postmenopausal osteoporosis. Report of a WHO Study Group. World Health Organ Tech Rep Ser..

[3] Tu KN, Lie JD, Wan CKV, Cameron M, Austel AG, Nguyen JK (2018). Osteoporosis: a review of treatment options. Pharm Ther.

[4] Burns ER, Stevens JA, Lee R (2016). The direct costs of fatal and non-fatal falls among older adults—United States. J Safety Res..

[5] Chen X, Wang Z, Duan N, Zhu G, Schwarz EM, Xie C (2018). Osteoblast-osteoclast interactions. Connect Tissue Res.

[6] Chan CKY, Mason A, Cooper C, Dennison E (2016). Novel advances in the treatment of osteoporosis. Br Med Bull.

[7] Chan KH, Zhuo S, Ni M (2013). Natural and synthetic peptide-based biomaterials for bone tissue engineering. OA Tiss Eng.

[8] Chan KH, Zhuo S, Ni M (2015). Priming the surface of orthopedic implants for osteoblast attachment in bone tissue engineering. Int J Med Sci..

[9] Yang D-H, Yang M-Y (2019). The role of macrophage in the pathogenesis of osteoporosis. Int J Mol Sci..

[10] Rao S-S, Hu Y, Xie P-L, Cao J, Wang Z-X, Liu J-H (2018). Omentin-1 prevents inflammation-induced osteoporosis by downregulating the pro-inflammatory cytokines. Bone Res..

[11] Pereira M, Petretto E, Gordon S, Bassett JHD, Williams GR, Behmoaras J (2018). Common signalling pathways in macrophage and osteoclast multinucleation. J Cell Sci.

[12] Vi L, Baht GS, Whetstone H, Ng A, Wei Q, Poon R (2015). Macrophages promote osteoblastic differentiation in vivo: implications in fracture repair and bone homeostasis. J Bone Miner Res.

[13] Glenske K, Donkiewicz P, Köwitsch A, Milosevic-Oljaca N, Rider P, Rofall S (2018). Applications of metals for bone regeneration. Int J Mol Sci.

[14] Balogh E, Paragh G, Jeney V (2018). Influence of iron on bone homeostasis. Pharmaceuticals.

[15] Amiri M, Salavati-Niasari M, Akbari A (2019). Magnetic nanocarriers: evolution of spinel ferrites for medical applications. Adv Colloid Interface Sci.

[16] McCarthy JR, Weissleder R (2008). Multifunctional magnetic nanoparticles for targeted imaging and therapy. Adv Drug Deliv Rev.

[17] Pistone A, Celesti C, Piperopoulos E, Ashok D, Cembran A, Tricoli A (2019). Engineering of chitosan-hydroxyapatite-magnetite hierarchical scaffolds for guided bone growth. Materials (Basel)..

[18] Pankhurst QA, Connolly J, Jones SK, Dobson J (2003). Applications of magnetic nanoparticles in biomedicine. J Phys D Appl Phys.

[19] Marycz K, Alicka M, Kornicka-Garbowska K, Polnar J, Lis-Bartos A, Wiglusz RJ (2019). Promotion through external magnetic field of osteogenic differentiation potential in adipose-derived mesenchymal stem cells: design of polyurethane/poly(lactic) acid sponges doped with iron oxide nanoparticles. J Biomed Mater Res Part B Appl Biomater..

[20] Blake RL, Hessevick RE, Zoltai T, Finger LW (1966). Refinement of the hematite structure. Am Miner.

[21] Shmakov AN, Kryukova GN, Tsybulya SV, Chuvilin AL, Solovyeva LP (1995). Vacancy ordering in γ-Fe_2_O_3_: synchrotron X-ray powder diffraction and high-resolution electron microscopy studies. J Appl Crystallogr.

[22] de Faria DLA, Silva SV, de Oliveira MT (1997). Raman microspectroscopy of some iron oxides and oxyhydroxides. J Raman Spectrosc.

[23] Jacob J, Abdul Khadar M (2010). VSM and Mössbauer study of nanostructured hematite. J Magn Magn Mater.

[24] Zboril R, Mashlan M, Petridis D (2002). Iron(III) oxides from thermal processes synthesis, structural and magnetic properties, Mössbauer spectroscopy characterization, and applications. Chem Mater.

[25] Lyubutin IS, Starchikov SS, Bukreeva TV, Lysenko IA, Sulyanov SN, Korotkov NYU (2014). In situ synthesis and characterization of magnetic nanoparticles in shells of biodegradable polyelectrolyte microcapsules. Mater Sci Eng C.

[26] Ramos Guivar JA, Bustamante A, Flores J, Mejía Santillan M, Osorio AM, Martínez AI (2014). Mössbauer study of intermediate superparamagnetic relaxation of maghemite (γ-Fe_2_O_3_) nanoparticles. Hyperfine Interact.

[27] Zakharova IN, Shipilin MA, Alekseev VP, Shipilin AM (2012). Mössbauer study of maghemite nanoparticles. Tech Phys Lett.

[28] Wu W, Xiao X, Zhang S, Peng T, Zhou J, Ren F (2010). Synthesis and magnetic properties of maghemite (γ-Fe_2_O_3_) short-nanotubes. Nanoscale Res Lett.

[29] The iron oxides: structure, properties, reactions, occurrences and uses, 2nd, completely revised and extended edition | Wiley [Internet]. Wiley.com. https://www.wiley.com/en-us/The+Iron+Oxides%3A+Structure%2C+Properties%2C+Reactions%2C+Occurrences+and+Uses%2C+2nd%2C+Completely+Revised+and+Extended+Edition-p-9783527606443. Accessed 30 Oct 2019.

[30] Strauch B, Patel MK, Navarro JA, Berdichevsky M, Yu H-L, Pilla AA (2007). Pulsed magnetic fields accelerate cutaneous wound healing in rats. Plast Reconstr Surg.

[31] Singh P, YashRoy RC, Hoque M (2006). Augmented bone-matrix formation and osteogenesis under magnetic field stimulation in vivo XRD, TEM and SEM investigations. Indian J Biochem Biophys.

[32] Patil S, Sandberg A, Heckert E, Self W, Seal S (2007). Protein adsorption and cellular uptake of cerium oxide nanoparticles as a function of zeta potential. Biomaterials.

[33] Zawisza K, Sobierajska P, Nowak N, Kedziora A, Korzekwa K, Pozniak B (2020). Preparation and preliminary evaluation of bio-nanocomposites based on hydroxyapatites with antibacterial properties against anaerobic bacteria. Mater Sci Eng, C.

[34] Beck-Speier I, Kreyling WG, Maier KL, Dayal N, Schladweiler MC, Mayer P (2009). Soluble iron modulates iron oxide particle-induced inflammatory responses via prostaglandin E2 synthesis: in vitro and in vivo studies. Particle Fibre Toxicol.

[35] Sadeghi L, Yousefi Babadi V, Espanani HR (2015). Toxic effects of the Fe_2_O_3_ nanoparticles on the liver and lung tissue. Bratisl Lek Listy.

[36] Zha L, He L, Liang Y, Qin H, Yu B, Chang L (2018). TNF-α contributes to postmenopausal osteoporosis by synergistically promoting RANKL-induced osteoclast formation. Biomed Pharmacother.

[37] Xia Y, Chen H, Zhang F, Wang L, Chen B, Reynolds MA (2018). Injectable calcium phosphate scaffold with iron oxide nanoparticles to enhance osteogenesis via dental pulp stem cells. Artif Cells Nanomed Biotechnol..

[38] Wang Q, Chen B, Ma F, Lin S, Cao M, Li Y (2017). Magnetic iron oxide nanoparticles accelerate osteogenic differentiation of mesenchymal stem cells via modulation of long noncoding RNA INZEB2. Nano Res..

[39] Yi C, Liu D, Fong C-C, Zhang J, Yang M (2010). Gold nanoparticles promote osteogenic differentiation of mesenchymal stem cells through p38 MAPK pathway. ACS Nano.

[40] Yang J, Zhang J, Ding C, Dong D, Shang P (2018). Regulation of osteoblast differentiation and iron content in MC3T3-E1 cells by static magnetic field with different intensities. Biol Trace Elem Res.

[41] Rodríguez JP, Montecinos L, Ríos S, Reyes P, Martínez J (2000). Mesenchymal stem cells from osteoporotic patients produce a type I collagen-deficient extracellular matrix favoring adipogenic differentiation. J Cell Biochem.

[42] Marędziak M, Marycz K, Lewandowski D, Siudzińska A, Śmieszek A (2015). Static magnetic field enhances synthesis and secretion of membrane-derived microvesicles (MVs) rich in VEGF and BMP-2 in equine adipose-derived stromal cells (EqASCs)-a new approach in veterinary regenerative medicine. Vitro Cell Dev Biol Anim..

[43] Marędziak M, Marycz K, Śmieszek A, Lewandowski D (2015). An in vitro analysis of pattern cell migration of equine adipose derived mesenchymal stem cells (EqASCs) using iron oxide nanoparticles (IO) in static magnetic field. Cell Mol Bioeng.

[44] Wu C, Chung AE, McDonald JA (1995). A novel role for alpha 3 beta 1 integrins in extracellular matrix assembly. J Cell Sci.

[45] Wang H, Li J, Zhang X, Ning T, Ma D, Ge Y (2018). Priming integrin alpha 5 promotes the osteogenic differentiation of human periodontal ligament stem cells due to cytoskeleton and cell cycle changes. J Proteomics..

[46] Hamidouche Z, Fromigué O, Ringe J, Häupl T, Vaudin P, Pagès J-C (2009). Priming integrin alpha5 promotes human mesenchymal stromal cell osteoblast differentiation and osteogenesis. Proc Natl Acad Sci USA.

[47] Shao P-L, Wu S-C, Lin Z-Y, Ho M-L, Chen C-H, Wang C-Z (2019). Alpha-5 integrin mediates simvastatin-induced osteogenesis of bone marrow mesenchymal stem cells. Int J Mol Sci..

[48] Vasiljeva O, Reinheckel T, Peters C, Turk D, Turk V, Turk B (2007). Emerging roles of cysteine cathepsins in disease and their potential as drug targets. Curr Pharm Des.

[49] Rachner TD, Khosla S, Hofbauer LC (2011). Osteoporosis: now and the future. Lancet.

[50] Hayman AR (2008). Tartrate-resistant acid phosphatase (TRAP) and the osteoclast/immune cell dichotomy. Autoimmunity..

[51] Lee M-S, Su C-M, Yeh J-C, Wu P-R, Tsai T-Y, Lou S-L (2016). Synthesis of composite magnetic nanoparticles Fe_3_O_4_ with alendronate for osteoporosis treatment. Int J Nanomed.

[52] Wang X, Chen B, Sun J, Jiang Y, Zhang H, Zhang P (2018). Iron-induced oxidative stress stimulates osteoclast differentiation via NF-κB signaling pathway in mouse model. Metab Clin Exp.

[53] Jia P, Xu YJ, Zhang ZL, Li K, Li B, Zhang W (2012). Ferric ion could facilitate osteoclast differentiation and bone resorption through the production of reactive oxygen species. J Orthop Res.

[54] Lötters FJB, van den Bergh JP, de Vries F, Rutten-van Mölken MPMH (2016). Current and future incidence and costs of osteoporosis-related fractures in the netherlands: combining claims data with BMD measurements. Calcif Tissue Int.

[55] Bukhari M (2009). The National Osteoporosis Guideline Group’s new guidelines: what is new?. Rheumatology (Oxford).

[56] Xu X-L, Gou W-L, Wang A-Y, Wang Y, Guo Q-Y, Lu Q (2013). Basic research and clinical applications of bisphosphonates in bone disease: what have we learned over the last 40 years?. J Transl Med..

[57] Amano S, Sekine K, Bonewald L, Ohmori Y (2009). A novel osteoclast precursor cell line, 4B12, recapitulates the features of primary osteoclast differentiation and function: enhanced transfection efficiency before and after differentiation. J Cell Physiol.

[58] Kornicka K, Marycz K, Tomaszewski KA, Marędziak M, Śmieszek A (2015). The effect of age on osteogenic and adipogenic differentiation potential of human adipose derived stromal stem cells (hASCs) and the impact of stress factors in the course of the differentiation process. Oxid Med Cell Longev..

[59] Schneider CA, Rasband WS, Eliceiri KW (2012). NIH image to ImageJ: 25 years of image analysis. Nat Methods.

[60] Chomczynski P, Sacchi N (1987). Single-step method of RNA isolation by acid guanidinium thiocyanate-phenol-chloroform extraction. Anal Biochem.

[61] Kornicka K, Szłapka-Kosarzewska J, Śmieszek A, Marycz K (2018). 5-Azacytydine and resveratrol reverse senescence and ageing of adipose stem cells via modulation of mitochondrial dynamics and autophagy. J Cell Mol Med..

[62] Livak KJ, Schmittgen TD (2001). Analysis of relative gene expression data using real-time quantitative PCR and the 2(−Delta Delta C(T)) Method. Methods.

