# Correction to: Iron oxides nanoparticles (IOs) exposed to magnetic field promote expression of osteogenic markers in osteoblasts through integrin alpha-3 (INTa-3) activation, inhibits osteoclasts activity and exerts anti-inflammatory action

**DOI:** 10.1186/s12951-020-00631-4

**Published:** 2020-05-19

**Authors:** K. Marycz, P. Sobierajska, M. Roecken, K. Kornicka-Garbowska, M. Kępska, R. Idczak, J.-M. Nedelec, R. J. Wiglusz

**Affiliations:** 1 The Department of Experimental Biology, University of Environmental and Life Sciences Wroclaw, Norwida 27B, 50-375 Wrocław, Poland; 2 Faculty of Veterinary Medicine, Equine Clinic-Equine Surgery, Justus-Liebig-University, Frankfurter 108, 35392 Giessen, Lahn, Germany; 3 Institute of Low Temperature and Structure Research, Polish Academy of Sciences, Okolna 2, 50-422 Wrocław, Poland; 4 International Institute of Translational Medicine, Jesionowa 11, Malin, 55-114 Wisznia Mała, Poland; 5 Université Clermont Auvergne, CNRS, SIGMA Clermont, ICCF, Clermont-Ferrand, France; 6 Centre for Advanced Materials and Smart Structures, Polish Academy of Sciences, Okolna 2, 50-950 Wrocław, Poland

## Correction to: J Nanobiotechnol (2020) 18:33 10.1186/s12951-020-00590-w

Following publication of the original article [1], the authors identified an omission in the ‘Funding’ section. The updated ‘Funding’ section is given below and the added statement has been highlighted in **bold typeface**.

Research was founded by National Science Centre Poland grant no: 2017/26/M/NZ5/01184. **The research is co-financed under the Leading Research Groups support project from the subsidy increased for the period 2020–2025 in the amount of 2% of the subsidy referred to Art. 387 (3) of the Law of 20 July 2018 on Higher Education and Science, obtained in 2019.**

## References

[1] Marycz K, Sobierajska P, Roecken M, Kornicka-Garbowska K, Kępska M, Idczak R, Nedelec JM, Wiglusz RJ (2020). Iron oxides nanoparticles (IOs) exposed to magnetic field promote expression of osteogenic markers in osteoblasts through integrin alpha-3 (INTa-3) activation, inhibits osteoclasts activity and exerts anti-inflammatory action. J Nanobiotechnol.

