# Supplementary material for: Iron oxides nanoparticles (IOs) exposed to magnetic field promote expression of osteogenic markers in osteoblasts through integrin alpha-3 (INTa-3) activation, inhibits osteoclasts activity and exerts anti-inflammatory action
Source: J Nanobiotechnology. 2020 Feb 18;18:33. doi: 10.1186/s12951-020-00590-w (PMC7027282; doi:10.1186/s12951-020-00590-w)
Supplement: Supplementary file 1 — Additional file 1. The list of the primers. [file 12951_2020_590_MOESM1_ESM.docx]

| **Gene** | **Primers (5’→3’)** | **Length of amplicon** | **Accession No.** |
| --- | --- | --- | --- |
| **Alp** | F: AACGTGGCCAAGAACATCATCA  R: TGTCCATCTCCAGCCGTGTC | 218 | NM_001287172.1 |
| **Bax** | F: AGGACGCATCCACCAAGAAGC  R: GGTTCTGATCAGCTCGGGCA | 251 | XM_011250780.3 |
| **Bcl2** | F: GGATCCAGGATAACGGAGGC  R: ATGCACCCAGAGTGATGCAG | 141 | NM_009741.5 |
| **Bmp-2** | F: GAGAACACCCGGAGAAGGAGG  R: AGCAGCCTCAACTCAAACTCG | 208 | NM_007553.3 |
| **Casp-9** | F: CCGGTGGACATTGGTTCTGG  R: GCCATCTCCATCAAAGCCGT | 278 | NM_001355176.1 |
| **Cat K** | F: TAACAGCAAGGTGGATGAAATCT  R: CTGTAGGATCGAGAGGGAGGTAT | 195 | XM_006500974.4 |
| **Cr1a** | F: TGCGGCGGGATCCTATAA  R: AGCCAGCAGTTGTCGTTGTA | 238 | NM_001355192.1 |
| **Col1a-1** | F: CAGGGTATTGCTGGACAACGTG  R: GGACCTTGTTTGCCAGGTTCA | 107 | NM_007742.4 |
| **Dmp-1** | F: CCCAGAGGCACAGGCAAATA  R: TCCTCCCCAATGTCCTTCTT | 211 | NM_001359013.1 |
| **Gapdh** | F: TGCACCACCAACTGCTTAG  R: GGATGCAGGGATGATGTTC | 177 | XM_017321385.2 |
| **Itga-1** | F: CACCTTTCAAACTGAGCCCGCCA  R: GCTGCCCAGCGATGTAGAGCACAT | 110 | NM_001033228.3 |
| **Itga-3** | F: TGGGCAAGTGCTATGTGCGTGGCA  R: TCTGGGTGAAGCCGCCGCTGGT | 147 | XM_006532311.4 |
| **Itga-6** | F: CTGGCTTCCTCGTTTGGCTATG  R: TGCCTTGCTGGTTAATGTAGACGT | 145 | XM_017315707.1 |
| **Itga-5** | F: ACAATGTAAGCCCAGTTGTGTCT  R: TTTGTAAGGCCACTGGAGATTTA | 236 | NM_008402.3 |
| **Itgb-1** | F: TCTCACCAAAGTAGAAAGCAGGGA  R: ACGATAGCTTCATTGTTGCCATTC | 138 | NM_010578.2 |
| **Itgb-3** | F: CTGCTCATCTGGAAGCTACTCAT  R: CACACACACACAAATTGTCCTCT | 233 | NM_016780.2 |
| **Mmp-9** | F: TTGCCCCTACTGGAAGGTATTAT  R: GAGAATCTCTGAGCAATCCTTGA | 172 | XM_006498861.3 |
| **Opn** | F: AGACCATGCAGAGAGCGAG  R: GCCCTTTCCGTTGTTGTCCT | 340 | NM_001204203.1 |
| **P21** | F: TGTTCCACACAGGAGCAAAG  R: AACACGCTCCCAGACGTAGT | 175 | NM_001111099.2 |
| **P53** | F: AGTCACAGCACATGACGGAGG  R: GGAGTCTTCCAGTGTGATGATGG | 287 | XM_030245924.1 |
| **Runx-2** | F: TCCGAAATGCCTCTGCTGTT  R: GCCACTTGGGGAGGATTTGT | 130 | NM_001271630.1 |
| **Sost** | F: AGCCTTCAGGAATGATGCCAC  R: CTTTGGCGTCATAGGGATGG | 134 | NM_024449.6 |
| **Trap** | F: GTCTCTGGGGGACAATTTCTACT  R: GTTTGTACGTGGAATTTTGAAGC | 241 | XM_006509945.3 |
| **c-fos** | F: CCAGTCAAGAGCATCAGCAA  R: TAAGTAGTGCAGCCCGGAGT | 248 | NM_010234.3 |
| **PU.1** | F: GAGAAGCTGATGGCTTGGAG  R: TTGTGCTTGGACGAGAACTG | 175 | XM_017316733.2 |
| **Tnf-a** | F: ACAGAAAGCATGATCCGCGA  R: CTTGGTGGTTTGCTACGACG | 295 | NM_013693.3 |
| **iNOS** | F: GACAAGCTGCATGTGACATC  R: GCTGGTAGGTTCCTGTTGTT | 325 | NM_001313922.1 |
| **Il-1b** | F: TGCCACCTTTTGACAGTGATG  R: TGATGTGCTGCTGCGAGATT | 138 | NM_008361.4 |
| **Tgfb1** | F: GGAGAGCCCTGGATACCAAC  R: CAACCCAGGTCCTTCCTAAA | 94 | NM_011577.2 |
| **Bglap** | F: CTCCTGAGAGTCTGACAAAGCCTT  R: GCTGTGACATCCATTACTTGC | 100 | NM_001032298.3 |
| **CaII** | F: TCAGGGAGCCCATTACTGTC  R: TCCAAATCACCCAGCCTAAC | 234 | NM_001357334.1 |

Alp- alkaline phosphatase; Bax- bcl-2-like protein 4; Bcl-2- B-cell lymphoma 2; Bmp-2- bone morphogenetic protein 2; Casp-9- caspase 9; Cat K- cathepsin K; Cr1a- calcitonin receptor isoform 1a; Col1a-1- alpha-1 type I collagen; dmp-1- dentin matrix acidic phosphoprotein 1; Gapdh- glyceraldehyde 3-phosphate dehydrogenase; Itga-1- integrin alpha 1; Intga-3- integrin alpha 3; Intga-6- integrin alpha 6; Intga-5- integrin alpha 5; Intgb-1- integrin beta 1; Intgb-3- integrin beta 3; Mmp-9- matrix metallopeptidase 9; Opn- osteopontin; p21- cyclin-dependent kinase inhibitor 1; p53- p53 tumor suppressor; Runx-2- runt-related transcription factor 2; Sost- sclerostin; Trap- tartrate-resistant acid phosphatase; c-fos- c-fos proto-oncogene; PU.1- transcription factor PU.1; Tnf-a- tumor necrosis factor α; iNOS- nitric oxide synthase; Il-1b- interleukin 1 beta; Tgfb-1- transforming growth factor beta 1; Bglap- osteocalcin; CAII- carbonic anhydrase II.
